# Joint-specific rheumatoid arthritis fibroblast-like synoviocyte regulation identified by integration of chromatin access and transcriptional activity

**DOI:** 10.1172/jci.insight.179392

**Published:** 2024-05-23

**Authors:** Eunice Choi, Camilla R.L. Machado, Takaichi Okano, David Boyle, Wei Wang, Gary S. Firestein

**Affiliations:** 1 Department of Chemistry and Biochemistry,; 2 Department of Medicine, and; 3 Department of Cellular and Molecular Medicine, UCSD, La Jolla, California, USA.

**Keywords:** Autoimmunity, Inflammation, Arthritis, Bioinformatics, Rheumatology

## Abstract

The mechanisms responsible for the distribution and severity of joint involvement in rheumatoid arthritis (RA) are not known. To explore whether site-specific fibroblast-like synoviocyte (FLS) biology might be associated with location-specific synovitis and explain the predilection for hand (wrist/metacarpal phalangeal joints) involvement in RA, we generated transcriptomic and chromatin accessibility data from FLS to identify the transcription factors and pathways. Networks were constructed by integration of chromatin accessibility and gene expression data. Analysis revealed joint-specific patterns of FLS phenotype, with proliferative, migratory, proinflammatory, and matrix-degrading characteristics observed in resting FLS derived from the hand joints compared with hip or knee. TNF stimulation amplified these differences, with greater enrichment of proinflammatory and proliferative genes in hand FLS compared with hip and knee FLS. Hand FLS also had the greatest expression of markers associated with an “activated” state relative to the “resting” state, with the greatest cytokine and MMP expression in TNF-stimulated hand FLS. Predicted differences in proliferation and migration were biologically validated with hand FLS exhibiting greater migration and cell growth than hip or knee FLS. Distinctive joint-specific FLS biology associated with a more aggressive inflammatory response might contribute to the distribution and severity of joint involvement in RA.

## Abstract 2

Integrated RNA-seq and ATAC-seq of rheumatoid arthritis fibroblast-like synoviocytes from different joint locations after culture in medium or TNF indicates joint-specific phenotypes contribute to the distribution and severity of joint inflammation.

## Graphical abstract

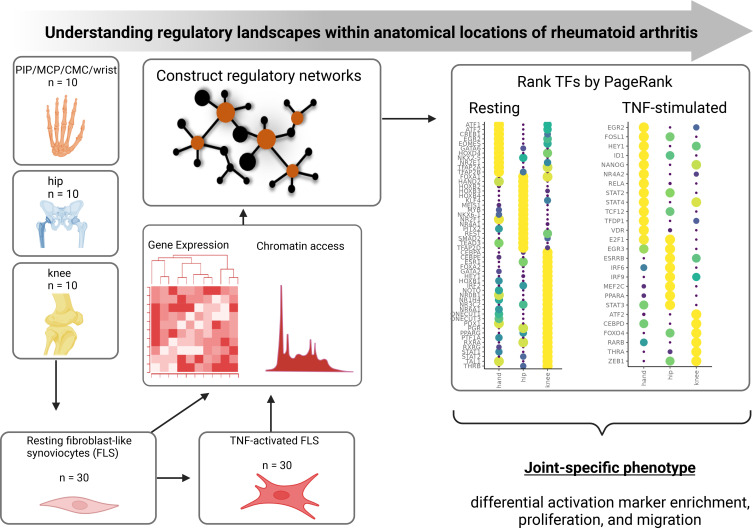

## Introduction

Rheumatoid arthritis (RA) is a common form of inflammatory arthritis characterized by autoimmunity and symmetric, polyarticular synovitis (1). RA fibroblast-like synoviocytes (FLS) are key effector cells in the synovium that are imprinted by their environment and display an aggressive phenotype (2, 3). For example, rheumatoid FLS produce a variety of proteases that damage the extracellular matrix and enable invasion of synovial tissue into bone and cartilage (2). These cells also produce cytokines such as IL-6 (1, 2, 4) and GM-CSF (2) that activate nearby immune effector cells like B and T lymphocytes (5, 6). Targeting FLS has been proposed as a strategy to avoid typical side effects like systemic immunosuppression associated with many current RA therapies (2).

RA is characterized by distinct anatomic and temporal patterns of joint involvement. The small joints of the hands and feet are often affected in very early RA; although larger joints like hips and knees can be affected early, joint damage in these locations typically occurs later in the course of disease. Biomechanics (7, 8), lymphatic distribution (9), and innervation (10) might contribute to the joint distribution in RA, but it is possible that location-specific FLS biology contributes (11–13). For example, we previously showed that FLS isolated from different joints have disease-independent and disease-specific methylation and transcriptomic patterns that are related to joint development and inflammatory pathways, respectively (13). Others have also shown that FLS from various joints have distinct characteristics and differential responses to the proinflammatory microenvironment (11, 12). Some of these might be related to increased expression of the long noncoding RNA (lncRNA) HOTAIR in knee FLS, although this only contributes to about half of the differences in hand FLS compared with knee and hip FLS (12). These data suggest that disease processes might vary from joint to joint and that the full extent of joint-specific regulatory networks needs to be defined.

To extend this work, RNA-seq and ATAC-seq of resting and TNF-stimulated FLS derived from 3 anatomical location in patients with RA were conducted to evaluate the FLS states in the stable and inflammatory contexts. We demonstrate that FLS from hand, knee, and hip have distinct transcriptomes and chromatin accessibility patterns. By integrating transcriptomes and epigenomes using our Taiji method (14, 15), we identified key transcription factor drivers that translate into unique joint-specific phenotypes, including predicted drivers that increase HOTAIR expression specifically in hand FLS. Moreover, joint-specific transcription factors (TFs) and regulatee chromatin accessibility and expression patterns correlate with patterns of joint involvement and severity in RA, namely with the greatest proinflammatory, proliferative, and migratory phenotype observed in hand FLS. Computational predictions were then biologically validated. These findings might contribute to the typical cadence of disease onset and progression in RA.

## Results

### Computational and experimental overview

**Figure 1:**
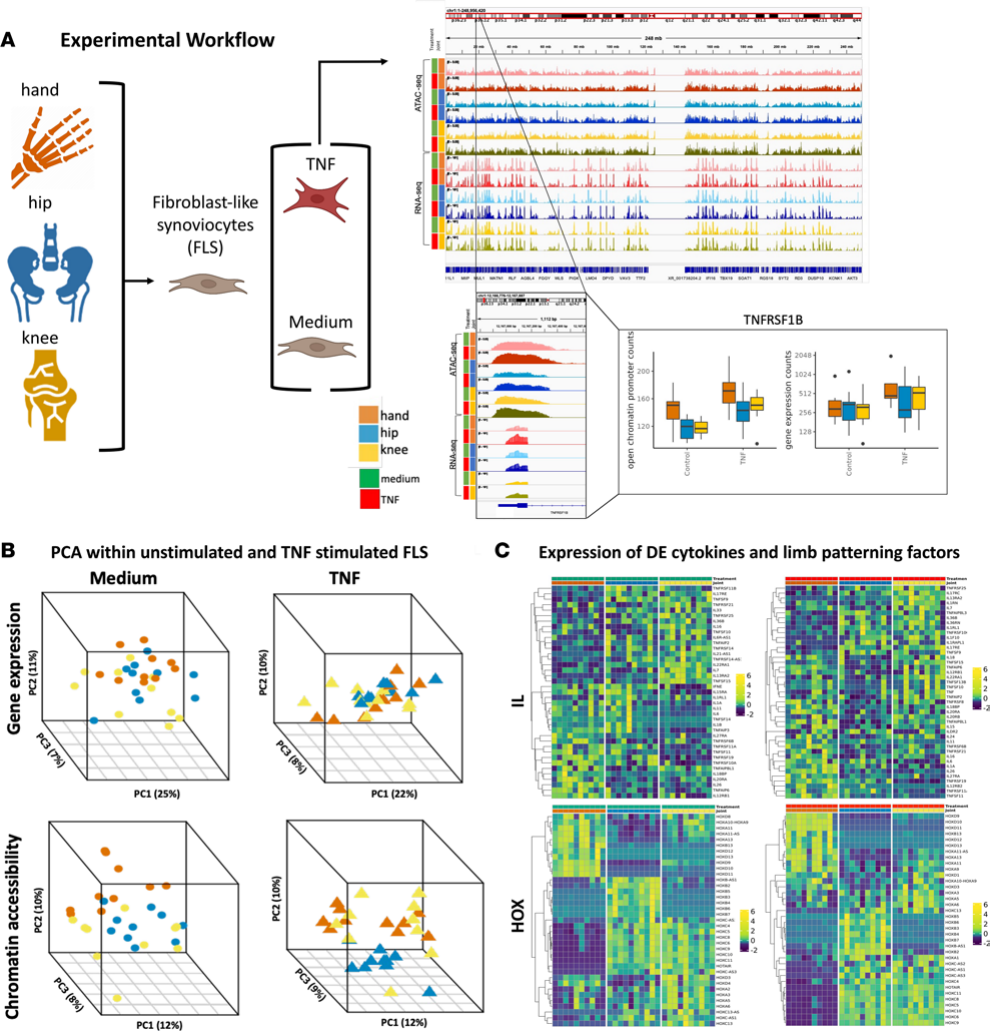
Experimental overview and stratification of RA FLS based on joint location. (**A**) Experimental workflow. Synovial tissue was obtained from RA patients at the time of clinically indicated arthroplasty at 3 joint locations: knee, hip, and hand (metacarpal phalangeal joint and wrist). Cells were cultured in medium or TNF-stimulated conditions and harvested for whole-genome RNA-seq and ATAC-seq. Amber, blue, and yellow represent hand, hip, and knee, respectively. Green and red represent medium and TNF-stimulated conditions, respectively. The color palette is maintained throughout all figures. (**B**) PCA of all gene expression and chromatin accessibility profiles within medium and TNF-stimulated conditions. PCA using the chromatin accessibility profiles revealed greater stratification between the joints compared to gene expression. (**C**) Gene expression profiles of differentially expressed interleukins and homeobox (HOX) genes. RA FLS have distinct cytokine expression in medium. Similarly, RA FLS have distinct patterns of cytokine induction after TNF stimulation. Several proinflammatory cytokines like IL-6 had marked induction in hand in response to TNF. Limb-patterning HOX gene expression was evaluated for medium and TNF-stimulated conditions. Hand FLS had the most distinct expression compared with hip and knee within both medium and TNF-stimulated conditions for several HOX features like HOXD10. See Supplemental Data Set 1 for DEGs, *P* values, and fold changes.

To test the hypothesis that FLS have distinct joint-specific phenotypes and behavior, we characterized FLS transcriptomes and chromatin accessibility in RA FLS obtained from synovial tissue isolated from 3 joint groups — 10 hands (7 metacarpal phalangeal [MCP] and proximal interphalangeal [PIP] joints, 1 first carpal metacarpal joint [CMC], 2 wrists), 10 hips, and 10 knees — from 30 different joints in 29 patients at the time of arthroplasty (see Methods). The cultured cells were studied in their basal condition as well as after TNF stimulation. Figure 1A shows the workflow for this process (see also Methods).

#### Stratifying RA FLS gene expression based on joint location.

Joint-specific transcriptional patterns were identified by first focusing our analysis on unstimulated cultured FLS. Principal component analysis (PCA) was performed on all gene expression profiles to summarize global patterns of gene expression and observed joint-specific differences, with the greatest overlap between hip and knee and the greatest separation between hand versus hip and hand versus knee (Figure 1B, top left PCA plot). To further define differences between joint-specific FLS, we conducted an additional PCA using pairwise differentially expressed genes (DEGs) for unstimulated FLS (Supplemental Data Set 1 and Supplemental Figure 1, left PCA plot; supplemental material available online with this article; https://doi.org/10.1172/jci.insight.179392DS1). This analysis revealed that hand joints are the most segregated from hip and knee along PC1 regardless of whether 2-way or 3-way analyses were performed. We then evaluated the PCA of gene expression profiles for TNF-stimulated FLS. Hand and knee FLS had the greatest variance and the greatest separation from hip FLS (Figure 1B, top right PCA plot). Again, an additional PCA using pairwise DEGs for TNF-stimulated FLS identified a stratification pattern similar to unstimulated FLS; along PC1, hand joints are the most segregated from hip and knee (Supplemental Figure 1, right PCA plot). Supplemental Data Sets 1 and 2 show the DEGs and their respective pathways between joint locations (hand-knee, hand-hip, and knee-hip) for unstimulated and TNF-stimulated FLS.

Because cytokines participate in the pathogenesis of RA (1, 2, 16), we focused our next analysis on cytokine expression patterns between joint locations for unstimulated and TNF-stimulated FLS. We have previously shown that IL-6 is differentially expressed and methylated between hip and knee FLS (13, 17). First, we evaluated joint-specific expression within unstimulated FLS (Figure 1C, top left plot). For cytokines that were differentially expressed in at least one pairwise comparison, we determined joint-specific expression patterns. FLS exhibited joint-specific expression of proinflammatory cytokines, with the greatest expression of cytokines observed in hand (16 cytokines), knee as intermediate (13 cytokines), and the least in hip (10 cytokines) (Figure 1C, top left plot). As a supplementary analysis, we performed hierarchical clustering using Ward’s hierarchical agglomerative clustering method (ward.d2) to determine expression patterns across joints. Unstimulated FLS exhibited joint-specific stratification, with the greatest mixing between hip and knee FLS (Supplemental Figure 2, left plot). We then evaluated joint-specific expression within TNF-stimulated FLS. After TNF stimulation, the pattern persisted with the greatest expression of differentially expressed cytokines observed in hand (21 cytokines), knee as intermediate (13 cytokines), and the least in hip (5 cytokines) (Figure 1C, top right plot). Again, we performed hierarchical clustering and found that TNF-stimulated hand FLS were clearly segregated from hip and knee FLS (Supplemental Figure 2, right plot). This reflects hand-specific FLS cytokine responses to TNF and is reflected by marked induction of several proinflammatory cytokines like IL-6 and IL-1α enriched in hand (Figure 1C, top right plot, and Supplemental Figure 2, right plot). These data suggest that RA FLS have distinct cytokine expression and responses to TNF based on joint location and might reflect RA severity.

We then evaluated joint-specific expression of limb-patterning genes, as they may contribute to defining joint-specific function and biology across RA patients (11–13). We evaluated expression of HOX genes between the FLS derived from hip, knee, and hand joints (Figure 1C, bottom left plot). First, we evaluated joint-specific expression for unstimulated FLS. For limb-patterning genes that were differentially expressed in at least one pairwise comparison within unstimulated FLS, we determined joint-specific expression patterns (Figure 1C, bottom left plot). Again, a supplementary hierarchical clustering analysis was conducted. We focused on HOX genes and identified topologically distinct gene expression patterns and determined the greatest mixing and gene expression similarity between hip and knee compared with hand (Supplemental Figure 3, left plot). As previously noted, FLS exhibited distinct joint-specific expression of several HOX genes (11–13) (Figure 1C, bottom left plot). The lncRNA HOTAIR was previously implicated as a regulator that contributes to these differences (12). Indeed, we confirmed that HOTAIR expression was greater in knee and hip FLS compared with hand FLS in unstimulated and TNF-stimulated conditions (Supplemental Figure 4A and Supplemental Figure 5). Our data suggest that these differences are likely related to decreased chromatin accessibility of HOTAIR regulatory regions in unstimulated hand FLS (Supplemental Figure 4B and Supplemental Figure 6.

Overall gene expression patterns were also evident under TNF-stimulated conditions, with consistently decreased expression in hand compared with hip or knee FLS (Figure 1C, bottom right plot). Again, we performed hierarchical clustering focused on HOX genes. After TNF stimulation, the topologically distinct patterns of HOX gene expression were still with greater similarity between hip and knee compared with hand FLS (Supplemental Figure 3, right plot).

#### Stratifying RA FLS chromatin accessibility based on joint location.

Previous studies determined distinct epigenetic patterns that reflect anatomic location, including differential methylation (13) and lncRNA (12) signatures. We conducted PCA on chromatin accessibility profiles with unstimulated-FLS data (Figure 1B, bottom left PCA plot). PCA revealed site-specific stratification that was more prominent than for the transcriptome. We then evaluated the PCA of chromatin accessibility profiles of TNF-stimulated FLS. (Figure 1B, bottom right PCA plot). All joint locations segregated from each other, but the greatest separation was observed between TNF-stimulated hip FLS compared with hand or knee FLS. Knee FLS also had greater mixing with hip FLS, while hand FLS had better separation from hip FLS. To evaluate potential effects of different hand surgical procedures or joint locations, a supplementary PCA using subcategorical joint location labels was used (Supplemental Figure 7). We did not observe stratification between wrist versus non-wrist hand samples using all epigenetic features. Supplemental Data Sets 2 and 3 show differential chromatin accessibility and the respective pathways between joint locations (hand-knee, hand-hip, and knee-hip) for unstimulated and TNF-stimulated FLS.

### Joint-specific patterns of unstimulated RA FLS transcriptional regulation

#### Unstimulated FLS have joint-specific transcriptional regulation.

**Figure 2:**
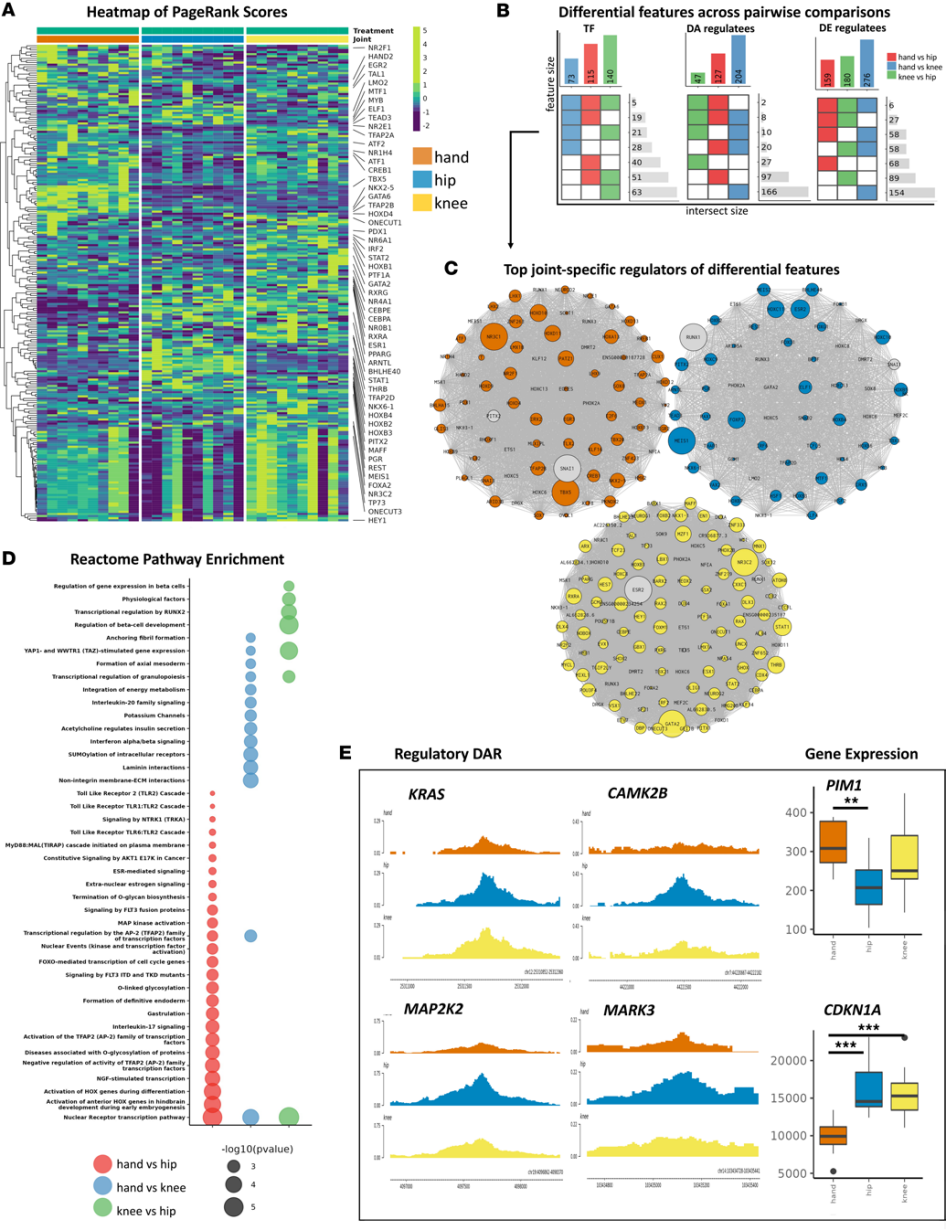
Joint-specific patterns of unstimulated RA FLS transcriptional regulation. (**A**) Heatmap of PageRanks of the top differential TFs selected from pairwise comparisons between unstimulated FLS from hand, hip, and knee. Pathway enriched TFs are labeled. For example, cAMP-responsive element–binding protein 1 (CREB1) has the greatest importance in hand FLS. Important TFs are reflected by increased accessibility of loci containing the TF motif, increased TF expression, and increased predicted TF binding affinity of the identified motif within the open chromatin regions. (**B**) Intersection plot of differential regulators (TFs) and regulatees (DARs and DEGs that are regulated by the differential regulator). Top bars indicate all differential features for each pairwise comparison. Heatmap indicates shared differential comparisons. Side bars indicate number of features that are shared between comparisons. For example, hand versus hip has 115 TFs that were identified with differential PageRanks. Fifty-one of those differential TFs were also identified from hip versus knee. Blue, red, and green are used for hand versus knee, hand versus hip, and knee versus hip comparisons, respectively. (**C**) For each joint, differential TFs with the greatest PageRank score in that joint was visualized. Nodes are scaled by PageRank. For example, PATZ1 has differential PageRanks between hand versus hip and has the greatest PageRank in hand (fold change in PATZ1 PageRank for hand versus knee: 1.2; hand versus hip: 1.3; knee versus hip: 0.8). (**D**) Pathway enrichment analysis of TFs and regulatee DARs and DEGs between pairwise comparisons. The greatest bias toward FLS aggressiveness was observed in hand versus hip comparisons and least between hip versus knee comparisons. Hand versus hip and hand versus knee comparisons were enriched for proinflammatory, e.g., “MAP kinase activation” (*P* < 0.003 by hypergeometric distribution test), and proliferative pathways e.g., “Constitutive signaling by AKT1 E17K in cancer” (*P* < 0.006 by hypergeometric distribution test). (**E**) Visualization of selected features. Genome browser shows differential open chromatin regulatory loci (left) and box-and-whisker plots are DEGs as representative examples of differential proliferative and proinflammatory features. The box-and-whisker plots display the median (line), the interquartile range (IQR) for the first (Q1) and third (Q3) quartiles (box bounds), and error bars (Q1 – 1.5 × IQR [low] and Q3 + 1.5 × IQR [high]). Values outside of the minimum and maximum are considered outliers (solid circles). **P < 0.01, ***P < 0.005 by Wilcoxon’s test.

Separate analysis of individual data sets like chromatin accessibility and the transcriptome provides some information on the functional differences in FLS based on joint location. However, to understand the distinct joint-specific networks in greater depth, ATAC-seq and RNA-seq data were integrated using our Taiji pipeline (14, 15) (see Methods). Taiji constructs networks using gene expression and open chromatin profiles and applies the PageRank score to all regulator nodes to quantify TF importance (see Methods). Using this method, we identified shared and joint-specific distinct TF PageRank scores in unstimulated FLS (Figure 2A). Using pairwise comparisons between joints, we identified 73, 115, and 140 differentially ranked TFs (Wilcoxon’s test, *P* value < 0.05) between hand versus knee, hand versus hip, and knee versus hip, respectively (Figure 2, A and B) (Supplemental Data Set 4). Hand FLS had increased PageRanks for TFs implicated in immune responses and inflammation, such as cytokine signaling, Toll-like receptor (TLR), and mitogen-activated protein kinase (MAPK) cascades (e.g., SMAD3, ATF1, ATF2, CREB1) (Figure 2C). Knee FLS were notable for the increased importance of TFs involved in IFN and NOTCH signaling (e.g., IRF2, STAT1, STAT2, HEY1, PBX1). Hip FLS, on the other hand, had increased importance of TFs focused on pathways like RUNX1 signaling (e.g., ELF1, LMO2, MYB) (Supplemental Data Set 4). These data indicate that FLS from the different joints have distinct regulators that drive differential FLS function and behavior.

#### Unstimulated FLS have joint-specific TF regulatee profiles.

We then evaluated joint-specific regulatees of these differential TFs in unstimulated FLS. To infer regulatory mechanisms of FLS phenotype, regulatees analyzed focused on DEGs or differentially accessible regions (DARs) (Figure 2B). Of interest, only limited overlap was observed between DEGs and DARs, likely because FLS were unstimulated and the regulatory regions are in an epigenetically poised rather than active state (Supplemental Figure 8). Evaluation of DEGs and DARs revealed distinct joint-specific drivers (Figure 2C). Pathway enrichment analysis of the union of the differential features (Figure 2B) revealed a gradient in FLS functional differences, with the greatest bias toward FLS aggressiveness between hand and hip and the least between hip and knee (Figure 2D). This gradient could reflect the pattern of disease-onset severity in joints involved in RA, which is often affected early in MCP and PIP joints and wrists. Hand versus hip and hand versus knee comparisons were enriched for proinflammatory and proliferative pathways in hand FLS, with minimal overlap for knee versus hip comparisons. Evaluation of DARs revealed FLS have joint-specific chromatin accessibility in key regulatory regions that confer well-characterized aggressive behaviors of RA FLS (2) (Figure 2E). For example, hand FLS had decreased expression of cell cycle inhibitors and increased expression of proto-oncogenes that are associated with cell growth (Figure 2E).

We also determined whether the potential regulator HOTAIR might contribute to joint-specific regulatory mechanisms in hand FLS (12). Due to high sample-specific heterogeneity in TF-HOTAIR edges, we applied a moderate filter of TF-HOTAIR edges that were identified in at least 20% of samples within each joint and treatment (unstimulated and TNF-stimulated). Taiji identified 0, 60, and 71 putative regulators of HOTAIR in unstimulated hand, hip, and knee, respectively, with 54 shared TFs between hip and knee (Supplemental Figure 9, left plot). TFs were ranked by TF-HOTAIR edge weights, which defines TF-gene regulatory potential to predict the most robust regulators that drive the hand FLS-specific phenotype. No predicted TFs for hand FLS were identified, which correlated with the lack of HOTAIR expression (Supplemental Figure 4A, Supplemental Figure 5). However, hip and knee FLS had distinct predicted regulators and TF-HOTAIR edge weights (Supplemental Figure 9, left plot). For example, hip and knee shared the top regulators (e.g., STAT1, STAT6, and MAZ), suggesting that similar regulatory mechanisms defined by epigenetics may drive increased HOTAIR expression in hip and knee FLS (Supplemental Figure 9, left plot).

### TNF amplifies joint-specific differences in RA FLS transcriptional regulation

#### FLS have joint-specific responses in transcriptional regulation.

**Figure 3:**
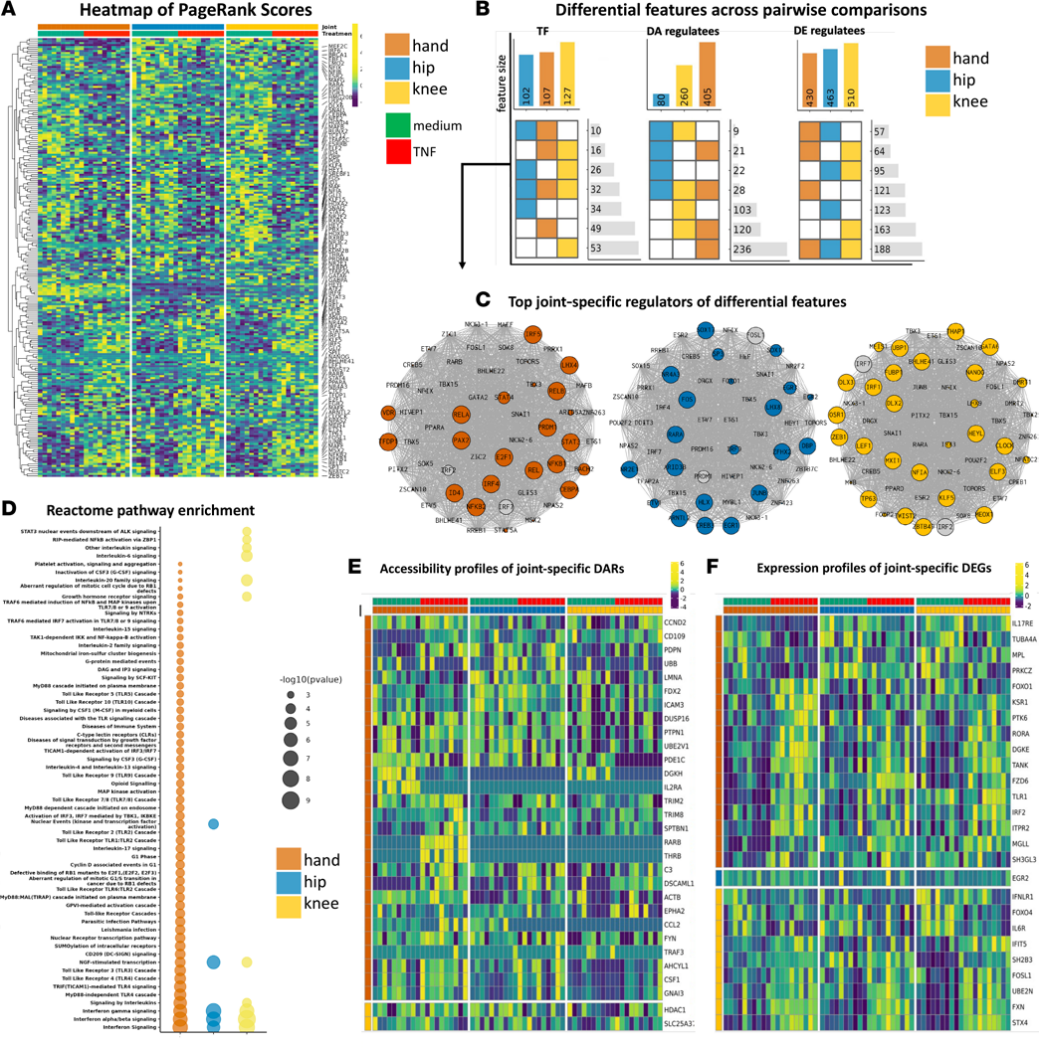
TNF amplifies joint-specific differences in RA FLS transcriptional regulation. (**A**) Heatmap of PageRanks of the top differential TFs selected from within joints and between unstimulated versus TNF-stimulated FLS. Hand FLS had unique increased importance of proliferative drivers, i.e., *E2F1*, *TFDP1*, and *FOXO4* and proinflammatory drivers, i.e., *STAT3*, *RREB1*, and *CEBPD*. See Supplemental Data Set 4 for differential TFs, *P* values, and fold change (FC) within each joint and between unstimulated versus TNF-stimulated FLS. (**B**) Intersection plot of differential regulators (TFs) and regulatees (DARs and DEGs that are regulated by the differential regulator). Top bars indicate all differential features for each pairwise comparison. Heatmap indicates shared differential comparisons. Side bars indicate number of features that are shared between comparisons. (**C**) For each joint, differential TFs between unstimulated versus TNF-stimulated FLS that regulate DEGs/DARs are visualized. Nodes are ranked by FC, i.e., large nodes indicate increased PageRank in TNF-stimulated versus unstimulated for that joint. Colored nodes have greatest FC in that joint. Gray nodes have greater FC in another joint (although it is still differential for that particular joint). (**D**) Pathway enrichment analysis of the union of TFs and regulatee DARs and DEGs between unstimulated versus TNF-stimulated FLS. Hand FLS exhibit unique enrichment of several proinflammatory, e.g., “MyD88-independent TLR4 cascade” (*P* < 1 × 10^–5^ by hypergeometric distribution test) and proliferative, e.g., “Aberrant regulation of mitotic cell cycle due to RB1 defects” (*P* < 0.002 by hypergeometric distribution test) pathways. (**E**) Joint-specific DARs that are pathway enriched between unstimulated versus TNF-stimulated FLS, e.g., genes that are only differentially accessible in one joint. Sum of all differentially accessible regulatory loci are visualized. Hand FLS exhibit the most dynamic chromatin accessibility in response to TNF. (**F**) Joint-specific DEGs that are pathway enriched between unstimulated versus TNF-stimulated FLS. Hand FLS exhibit the most aggressive and joint-specific transcriptomic changes in response to stimuli.

Joint-specific responses in TF PageRanks were then evaluated by comparing unstimulated versus TNF-stimulated FLS within each joint. As expected, proinflammatory TFs like *NFKB1*, *NFKB2*, and *RELB* had significantly increased PageRanks after TNF stimulation for FLS derived from all joint locations (Figure 3A and Supplemental Data Set 4) (Wilcoxon’s test, *P* < 0.05). Among the shared differential TFs, hand FLS compared with other joints had increased induction of proinflammatory TFs, such as *RELA*, *RELB*, and *NFKB2*. Furthermore, hand FLS had unique differential TF PageRanks associated with proliferation, such as *E2F1*, *TFDP1*, and *FOXO4* and proinflammatory drivers like *STAT3*, *RREB1*, and *CEBPD* (Supplemental Data Set 4). The observations suggest that TNF-stimulated hand FLS have epigenetic and transcriptomic responses that disproportionately enhance proinflammatory and proliferative TF functions compared with knee or hip FLS.

#### TNF amplifies joint-specific differences in regulatee profiles.

The regulatees of differential TFs were then evaluated after TNF stimulation, again focusing on DEGs and DARs (Figure 3B). TNF-stimulated FLS had joint-specific TFs and that reflected site-specific epigenetic and transcriptomic responses (Figure 3C). Pathway enrichment of the union of all differential features (Figure 3B) again revealed a gradient in proinflammatory responses, with the greatest and least proinflammatory pathways observed in hand and hip, respectively, with knee as intermediate (Figure 3D). For example, hand FLS had unique enrichment of proliferative pathways like “G1 Phase” and “Cyclin D–associated events in G1” (Figure 3D and Supplemental Data Set 5). Evaluation of joint-specific and pathway enriched DARs revealed hand FLS might also have significant epigenetic plasticity (Figure 3, B and E) that correlates with hand-specific differential PageRanks in chromatin-remodeling TFs, such as *CTCF*, *ATF2*, *KDM2B*, *SPI1*, and *TFAP2C* (Wilcoxon’s test, *P* < 0.05). Furthermore, evaluation of pathways enriched and their unique DEGs revealed that hand FLS have the greatest expression of proinflammatory and proliferative genes (Figure 3F). As with our analysis of unstimulated FLS, decreased HOTAIR chromatin accessibility was observed (Supplemental Figure 4B and Supplemental Figure 6). We identified key regulators of HOTAIR after TNF stimulation and identified 0, 22, and 36 putative regulators of HOTAIR for hand, hip, and knee, respectively, and identified the drivers in the latter 2 included proinflammatory TFs like *NFKB1*, *RELB*, and *IRF1* (Supplemental Figure 9, right plot). This suggests a more complex mechanism to explain joint-specific response to TNF and clarifies the role of HOTAIR in the context of each transcriptional network that drives joint-specific FLS function. From the evaluation of joint-specific differences in PageRanks, hand-specific differences observed in unstimulated FLS were amplified by TNF and reflected by greater numbers of joint-specific pathways and transcriptomic and epigenomic responses compared with hip and knee FLS.

### Validation of computational predictions

#### Hand FLS have significant enrichment of genes associated with an “activated” state.

**Figure 4:**
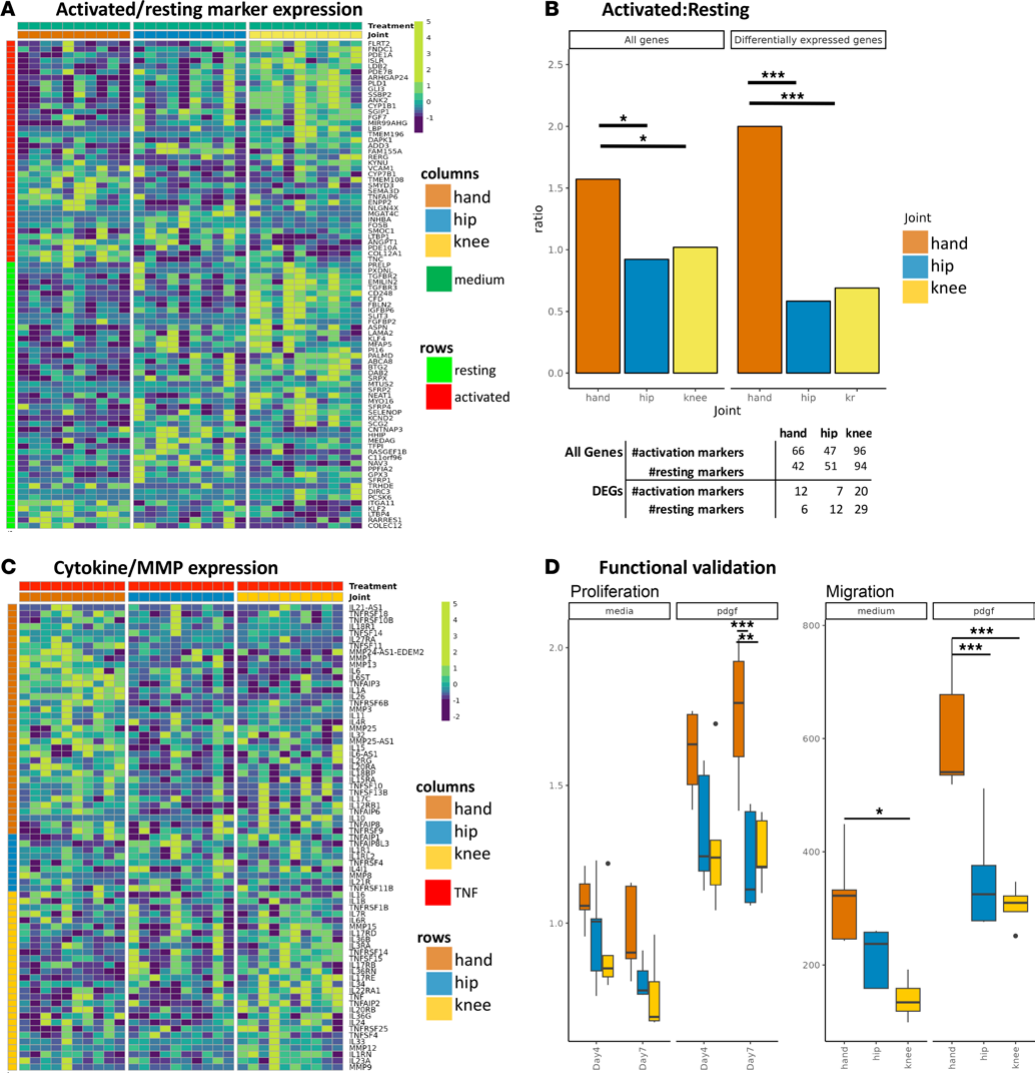
Validation of computational predictions. (**A**) Heatmap of differentially expressed activated and resting marker expression in unstimulated FLS. Rows are activated/resting markers. While both hand and knee have increased expression of activated markers, knee also has increased expression of resting markers, indicating hand FLS have the greatest ratio of activated to resting marker expression. (**B**) Ratio of number of markers of maximum expression within each joint. Using either all markers or differentially expressed markers revealed hand FLS have a significantly increased “activated” to “resting” marker ratio (20). *P < 0.05, ***P < 0.005 by permutation test. The table indicates the number markers with greatest expression in that joint. For example, using all markers, hand FLS had the greatest expression of 66 activated and 42 resting markers. Hand FLS retain the greatest ratio when limiting markers to DEGs. (**C**) Heatmap of differentially expressed cytokines and MMPs in TNF-stimulated FLS. Rows indicates what joint has the greatest expression of that gene. TNF-stimulated hand FLS have the greatest number of cytokines and MMPs with greatest expression compared with the other joints. (**D**) Proliferation and migration biologic validation. Unstimulated and PDGF-stimulated hand FLS proliferate faster than hip or knee FLS. We also evaluated differences in unstimulated and PDGF-stimulated migration and found that hand FLS exhibited greater migration capacity than hip or knee FLS. *P < 0.05, **P < 0.01, ***P < 0.005 by 1-way ANOVA followed by Holm-Šidák multiple-comparison test.

Recent studies of fibroblast states in RA synovium showed that these cells can exist in “activated” and “resting” states (18). Given our prediction of increased pathogenicity of hand FLS, we tested whether hand FLS are enriched for genes associated with activated FLS states as defined by that report. Hand and knee had increased expression of activation state markers; however, knee FLS also had enrichment of resting markers (Figure 4A). To clarify the cell states for FLS from different joint locations, we evaluated the ratio of activated to resting genes for each joint. We found that hand FLS have a significantly increased ratio of activation markers compared with hip or knee FLS using either DEGs or all markers (permutation test, *P* < 0.05) (Figure 4B, Supplemental Figure 10, and Supplemental Figure 11). These data suggest that the RA hand synovium is enriched for FLS in the activated state and is consistent with our prediction that hand FLS are more likely to be aggressive.

#### Joint-specific response to TNF reflects differential cytokine and MMP signaling.

We then determined the cytokine and MMP expression in TNF-stimulated FLS to assess whether those genes reflect the responses associated with the activated fibroblast state. We first evaluated cytokines and MMPs that were differentially expressed between unstimulated and TNF-stimulated FLS and determined the TNF-stimulated FLS joint with the greatest expression (Figure 4C). Hand and hip FLS had the greatest and least upregulation of cytokines and MMPs, respectively (Figure 4C). The burden of cytokine and MMP gene expression was determined and showed that hand FLS had significantly increased cumulative induction when using either differentially expressed or all cytokines and MMPs (Supplemental Figure 12 and Supplemental Figure 13) (permutation test, *P* < 0.05).

#### Increased growth and migration of RA hand FLS.

Our computational predictions also suggested that hand FLS might be more aggressive and exhibit differential proliferation and migration compared with hip and knee FLS. Potential differences in proliferation were biologically validated using a cell growth assay, which showed that unstimulated and PDGF-stimulated hand FLS proliferate faster than hip or knee (1-way ANOVA, *P* < 0.05) (Figure 4D). We also biologically tested differences in unstimulated and PDGF-stimulated migration and found that hand FLS migration was greater than that of hip or knee FLS (Figure 4D).

## Discussion

RA is a symmetric polyarticular arthritis with a characteristic joint distribution. The small joints of the hands, especially the MCP joints, are often affected first and can be the most symptomatic. Although there is variability in the clinical course, the disease often progresses to involve larger joints like knees and hips. The mechanisms that explain the distribution of joints in RA are not understood and could relate to the unique biomechanics of each joint (7, 8) or other intrinsic factors. Alternatively, synovial cells might be programmed with joint-specific functions that can participate in embryonic development (19) as well as contribute to the RA clinical phenotype.

Our epigenetic studies of synovial inflammation in RA have focused on FLS as key stromal elements that can shape the joint structure and function (20, 21). These cells display a unique aggressive phenotype in RA that contributes to synovial inflammation and joint damage (2, 18, 22). Interestingly, they can potentially spread disease through migration of activated fibroblasts between joints (22). The role of fibroblasts was also supported by data demonstrating an increase in circulating mesenchymal cells known as PRIME cells prior to RA disease flares (23). Our understanding of FLS biology was advanced by the discovery of aberrant epigenetic marks involving genes and pathways associated with inflammation and cell migration and invasion in cultured RA FLS (11, 20, 21, 24). It is not clear whether these marks are imprinted before cells leave the bone marrow or whether this occurs in situ after cells have migrated to the synovium.

In addition to these overall RA-related epigenomic changes in FLS, marks that are specific to individual joint locations have also been described. Those data largely focused on DNA methylation and identified 2 types marks: (a) disease-independent epigenetic marks that are found mainly in genes like the homeobox family and likely play a role in embryonic development, and (b) disease-specific marks involving genes and pathways that participate in inflammation and distinguish FLS isolated from different joint locations (11, 13). The lncRNA HOTAIR has also been implicated as one mechanism for joint-specific gene expression (12). Its effects are limited to a subset of hand-knee differences and are not likely responsible for knee-hip differences. Moreover, our analysis reveals that the regulatory network is likely responsible for the lack of HOTAIR expression in hand FLS and that a much broader regulatory network beyond that lncRNA is defined by the joint of origin. To define these interactions, we generated a new data set that compares features of resting and TNF-stimulated FLS derived from hands, knees, and hips and analyzed it using an algorithm that integrates transcriptome and chromatin accessibility. Distinctive joint-specific patterns of TF and regulatees suggest that hand FLS are broadly programmed to be more aggressive than FLS derived from knees or hips, including an array of poised enhancers and promoters that can be activated by TNF. These observations, along with biological validation of computational predictions, suggest that FLS imprinting contributes to RA clinical phenotype. The Taiji method previously showed that patients could be stratified into biologically distinct groups based on their individual FLS biology and TF function (14, 15). This method integrates subtle differences between thousands of transcriptional and chromatin accessibility features that are globally summarized by PageRank scores of each TF. Evaluation of differential TFs and regulatees reflect the inherent epigenetic, transcriptomic, and TF differences that synergistically drive joint-specific FLS behavior. As noted above, the method has advantages over analyzing individual data sets like RNA-seq or ATAC-seq. For example, the Taiji networks revealed unique inflammatory (e.g., TLR1/2 cascades), proliferative (e.g., FOXO-mediated transcription of cell cycle genes), and matrix-related (e.g., laminin interactions) pathways that were not identified with DEGs or DARs alone (Supplemental Data Set 2). This work demonstrates the potential for integrative analyses to identify relevant pathways and therapeutic targets.

Unstimulated cultured RA FLS had transcriptomic and epigenetic patterns that distinguished between joints, as previously reported (11, 13). Focused unsupervised clustering using only cytokines or limb development factors identified a similar pattern, with greatest similarity of transcriptomic patterns shared between hip and knee that was distinct from hand FLS. These observations are consistent with other recent studies where hand and shoulder FLS were distinct from knee (11). Integration of our data by Taiji identified FLS derived from hand had enrichment of proliferative and proinflammatory regulatees of the TFs with the highest PageRank. Epigenetic differences had amplified enrichment of proliferative and inflammatory features in chromatin accessibility that were not yet reflected by the transcriptome, indicating the FLS from the different joints may be epigenetically poised.

Perhaps the most notable results showed that differences between joints were amplified with TNF. FLS derived from hands, which already had features that correlate with more aggressive behavior, were distinguished by further enhancement of proinflammatory, proliferative, and matrix-destructive genes compared with the other joints. These data are consistent with other recent studies that have shown that hand fibroblasts have greater MMP13 expression after TNF stimulation (11). Joint-specific sensitivity of FLS and clinical responses in RA to methotrexate and tofacitinib have been observed (17, 25), and it is also possible that responses to TNF inhibitors could be influenced, in part, by joint location. In addition, our study suggests that hand FLS display the greatest global epigenetic plasticity based on differential regulation of key chromatin remodelers and the greatest global epigenetic changes in response to TNF. It is not certain whether these marks are pre-programmed or whether different mechanical loads modulate distinct inflammatory signaling (26, 27) and epigenetic changes (28). Thus, the mechanisms of imprinting are largely unknown.

Taiji-identified hand FLS are fundamentally distinct from hip and knee in medium and TNF-stimulated conditions. We used the recently reported “activated” and “resting” markers (18, 29) to corroborate our computational predictions in FLS severity and aggressiveness between the joints. We found that unstimulated hand FLS had significant transcriptomic upregulation of “activated” genes and lowest expression of “resting” genes, with marked upregulation of cytokines and MMPs in TNF-stimulated hand FLS. This observation agrees with previous work that reports greatest expression of cytokines/MMPs in “activated” cells (18). Unstimulated and TNF-stimulated FLS also had distinct expression and chromatin accessibility profiles of genes and pathways implicated in proliferation and migration. Our biologic validation studies also confirmed that hand FLS had displayed increased proliferation and migration compared with hip and knee FLS.

Our work does have some limitations. Age, sex, and type of surgical procedure have potential confounding effects, but our cohorts were well matched for these variables (see Methods). As our data are largely derived from deidentified samples, we are unable to determine disease activity using clinical metrics. However, the population was homogeneous in that all patients, including the wrist surgeries, had longstanding, severe RA requiring arthroplasty. Evaluation of other anatomical locations would also be of interest. In the present study, we focused on the 3 joint locations with the most common surgeries to have sufficient material (hand, hip, and knee). Although we focused on late RA with substantial joint damage, it is possible that earlier disease might have distinct mechanism and epigenetic marks (30). Despite these limitations, we were able to identify significant enrichment of the activated state signature recently identified in lining and sublining RA fibroblasts (18). Synovial fibroblasts cultured from synovium (conventional FLS) converge to a common phenotype in culture (15), so the relationship between the in vivo and in vivo categories is still uncertain. However, the correlation between the active state and our integrated analysis is striking and suggests that it is relevant to cultured cells.

In conclusion, we performed an integrative genomic analysis that identifies joint-specific differences in FLS biology. The correlation with distribution of joints and the cadence of joints as RA progresses is intriguing and reiterates the importance of joint context in synovial biology. This, along with other influences like biomechanics, lymphatic distribution, and innervation, could contribute to the distribution of joint involvement in RA. Further investigation into features that shape topological potential for inflammation might provide insight into joint-specific pathways that could guide therapy.

## Methods

### Sex as a biological variable.

Sex was not a criterion for patient selection in this study. There was a greater number of females than males in each group (hip, knee, hand) due to the higher prevalence of RA in females. Frequency of sex was consistent across joints, with 1, 2, and 2 male and the rest female within each group for hand, hip, or knee, respectively.

### Synovial tissue and FLS.

Synovial tissue was obtained from RA patients at the time of clinically indicated arthroplasty from 3 joint locations to generate 30 FLS lines: knee (10 patients), hip (10 patients), and hand (9 patients from PIP, MCP, CMC, and wrist (1 patient had a sample from an MCP and another sample from a PIP). Patient ages were similar across joint locations (hand, 57.8 ± 10.3; hip, 61.2 ± 13.1; knee, 59.3 ± 7.9 years; pairwise Wilcoxon’s test *P* > 0.05 for all pairwise joint locations). The diagnosis of RA conformed to American College of Rheumatology 2010 criteria (31). Patients were homogeneous in that all had significant joint damage with longstanding disease requiring major surgical intervention. For FLS derived from hand, 7 of 10 had MCP and PIP arthroplasties, 1 had first CMC arthroplasty, and 2 had wrist arthroplasty, including tendon repair and distal ulna resection. The synovium was processed as previously described (15, 32); briefly, cells were cultured in Dulbecco’s modified Eagle’s medium (DMEM; Life Technologies) supplemented with 10% heat-inactivated fetal calf serum (FCS) (Gemini Bio-Products), and supplements (penicillin, streptomycin, gentamicin, and glutamine) in a humidified atmosphere containing 5% CO_2_. Cells were allowed to adhere overnight, and then nonadherent cells were removed. Adherent FLS were split at 1:3 when they were 70%–80% confluent and used from passages 4 through 7. Cultures at 70%–80% confluence were exposed to media or 50 ng/mL of TNF for 6 hours the cells were harvested and processed for ATAC-seq or RNA-seq.

### RNA-seq and ATAC-seq sample processing and assays.

Genomic DNA and total RNA from parallel FLS cultures were used for RNA-seq and ATAC-seq. Total RNA was extracted and the quality of all samples was evaluated using an Agilent Bioanalyzer. The samples had an average RNA integrity number (RIN) of 9.4, with a minimum of 8. Sequencing libraries were prepared using TruSeq Stranded Total RNA RiboZero protocol from Illumina. Libraries were pooled and sequenced with an Illumina HiSeq 2000 (Institute for Genomic Medicine, UCSD). Genomic DNA was processed for ATAC-seq (Center for Epigenomics, UCSD) and subsequently sequenced (Institute for Genomic Medicine, UCSD).

### RNA-seq and ATAC-seq data processing.

To facilitate reproducible results, RNA-seq and ATAC-seq fastq files were processed using the nf-core pipeline (33), which verifies raw sequencing quality, trims adapters, removes genome contaminants, removes ribosomal RNA, deduplicates, and conducts additional extensive quality control for RNA-seq. For ATAC-seq, nf-core pipeline verifies raw sequencing quality, trims adapters, deduplicates, removes mitochondrial DNA, blacklisted regions, low-quality unmapped, multi-mapped, mismatched, or multi-chromosomal-matched alignments, and conducts additional extensive quality control. No data were excluded from RNA-seq or ATAC-seq analysis for quality reasons. Environment for nf-core/RNA-seq and nf-core/ATAC-seq was as follows: singularity v3.8.6–1.e18, nextflow v22.04.0, with either nf-core/rnaseq v3.8.1 or nf-core/atacseq v1.2.1 with all default nf-core parameters. Briefly regarding the RNA-seq processing workflow, reads were trimmed with Trim Galore! v.0.6.7 (http://www.bioinformatics.babraham.ac.uk/projects/trim_galore/Accessed June 15, 2018.) aligned to hg38 with STAR v2.7.10a, and bam-level quantification performed with salmon v0.13.1. For ATAC-seq, reads were trimmed (as above), aligned to hg38 with BWA v0.7.17, blacklisted regions (37) were filtered, and narrow peaks were called with macs2 v2.2.7.1 (38). Only autosomes were used for downstream analysis. RNA-seq was sequenced with only 1 batch. ATAC-seq was sequenced with 2 batches. No significant batch effects were observed (Supplemental Figure 14). All DEGs were found with the nonparametric Wilcoxon’s test (*P* < 0.05, log_2_[fold change] > 0.58). All DAR analysis (FDR < 0.05, log_2_[fold change] > 0.58) was conducted with the DiffBind R package (39).

### Construction of genetic networks using Taiji.

We used the Taiji (14, 15) algorithm (v.1.3.0) to incorporate RNA-seq, ATAC-seq, and enhancer-promoter interactions (the top 10% most confident predictions from Epitensor; ref. 40) (Supplemental Figure 15). For each sample, Taiji produced a genetic network and inferred TF importance via personalized PageRank calculations for 745 TFs with known motifs. Briefly, the nodes in the network are genes and are weighted by the normalized gene-expression level. To create edges between regulators and regulatees, open chromatin promoter and enhancer regions are queried for TF motifs documented in the Cis-BP database (41). These edges are weighted by the motif score reflecting the binding affinity, TF expression levels, and target open chromatin peak intensity. The personalized PageRank algorithm is then applied to the resulting directional and weighted network to determine relative importance of each TF for each sample. The output of the Taiji workflow for each sample are the personalized PageRank scores for 745 TFs, the network topology consisting of directional regulator-regulatee relationships with each pair’s associated edge weight and node weights. Individual genetic networks were constructed for 30 RA FLS cell lines derived from various anatomical locations (hand, knee, hip) and conditions (unstimulated, TNF stimulated) for 60 total samples. Most confident regulator-regulatees edges (shared by >70% samples within joint and condition) were used for downstream analysis (Supplemental Data Set 6).

### Proliferation and migration assays.

For proliferation, FLS were plated in 96-well plates, 3000 cells/well in quadruplicate, as described above. Cells were serum starved with 1% FCS for 24 hours followed by incubation with 1% FCS with and without PDGF (10 ng/mL). MTT (20 μL; Invitrogen) was added to each well and the plates read at 550 nm, using 690 nm as reference. For migration, RA FLS were plated into 12-well plates (1.5 × 10^5^ cells/well) and starved with 0.1% FCS/DMEM and a scratch introduced with a pipette tip. After culturing for 24 hours in the presence or absence of PDGF (10 ng/mL) in 0.1% FCS/DMEM, cells were fixed with 4% paraformaldehyde and stained with crystal violet. They were visualized using an ECLIPSE E800 (Nikon) microscope at ×40 magnification and counted with ImageJ (NIH).

### Permutation test.

Significant enrichment of activation markers between joints *i* and *j* were identified using a permutation test by randomly shuffling the joint labels with the greatest expression (defined by the median for each joint) of that activated/resting marker and then recalculating the ratio of the number of maximally activated markers to the number of resting markers. The test statistic is defined as *P* = *Ratio_i_* – *Ratio_j_*.

We also identified enrichment of cumulative cytokine expression in TNF-stimulated cell lines by taking the cumulative expression of cytokines and MMPs averaged (by median) per joint. For each pairwise comparison, significant upregulation of cytokines between joints *i* and *j* were identified using a permutation test by randomly shuffling the joint labels and taking the sum expression *P* = sum(cytokine/MMP)*_i_* – sum(cytokine/MMP)*_j_*. For both permutation tests, the *P* value was computed by *P* = {#*m*|*P*(*i*′*,j*′) < *P*(*i,j*), *m* = 1, 2…*M*}/*M*, where *m* is each permutation, *M* = 5000 total permutations, and #*m* is the number of permutations that fulfill the test statistic, e.g., *P*(*i*′*,j*′) < *P*(*i,j*).

### Statistics.

All statistical tests were conducted with the nonparametric Wilcoxon’s test unless otherwise specified. A *P* value of less than 0.05 was considered significant. No samples were excluded from this analysis.

### Study approvals.

These studies were approved by the UCSD Institutional Review Board (IRB 014-0175). All research participants signed informed consent.

### Data availability.

The data that support the findings of this study are available in dbGaP (accession phs003633.v1.p1) and in the supplemental Supporting Data Values file. All code used for downstream analysis is provided in https://github.com/ejc043/TaijiRAJoint/

## Author contributions

EC performed analyses and wrote the manuscript. CRLM and TO collected data and conducted experiments. DB designed the research studies, collected data, and wrote the manuscript. WW and GSF designed the research studies and wrote the manuscript.

## Supplementary Material

Supplemental data

Supplemental data set 1

Supplemental data set 2

Supplemental data set 3

Supplemental data set 4

Supplemental data set 5

Supplemental data set 6

Supporting data values

## Version 1 05/23/2024

In-Press Preview

## Version 2 06/24/2024

Electronic publication
