# Supplementary material for: Joint-specific rheumatoid arthritis fibroblast-like synoviocyte regulation identified by integration of chromatin access and transcriptional activity
Source: JCI Insight. 2024 May 23;9(12):e179392. doi: 10.1172/jci.insight.179392 (PMC11383168; doi:10.1172/jci.insight.179392)
Supplement: Supplemental data [file jciinsight-9-179392-s232.pdf]

Supplementary figures

S1. PCA using differentially expressed genes within Control (left) and TNF (right) stimulated FLS.

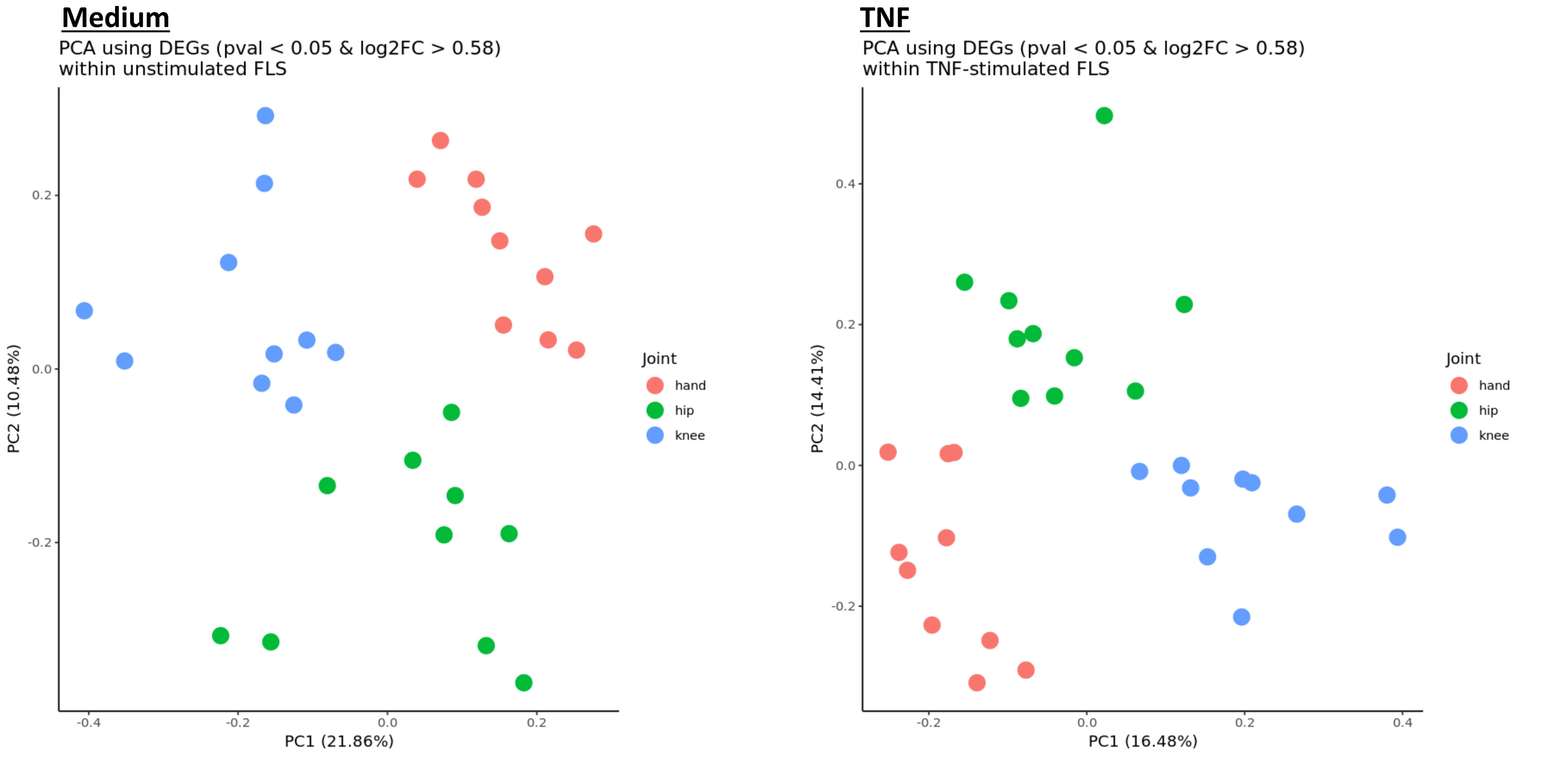

S1. PCA using differentially expressed genes within Control (left) and TNF (right) stimulated FLS. Within unstimulated FLS, hip and knee FLS have the greatest overlap across PC1. Similar stratification patterns are observed within TNF-stimulated FLS despite using all pairwise DEGs.

S2. Unsupervised clustering using differentially expressed cytokines

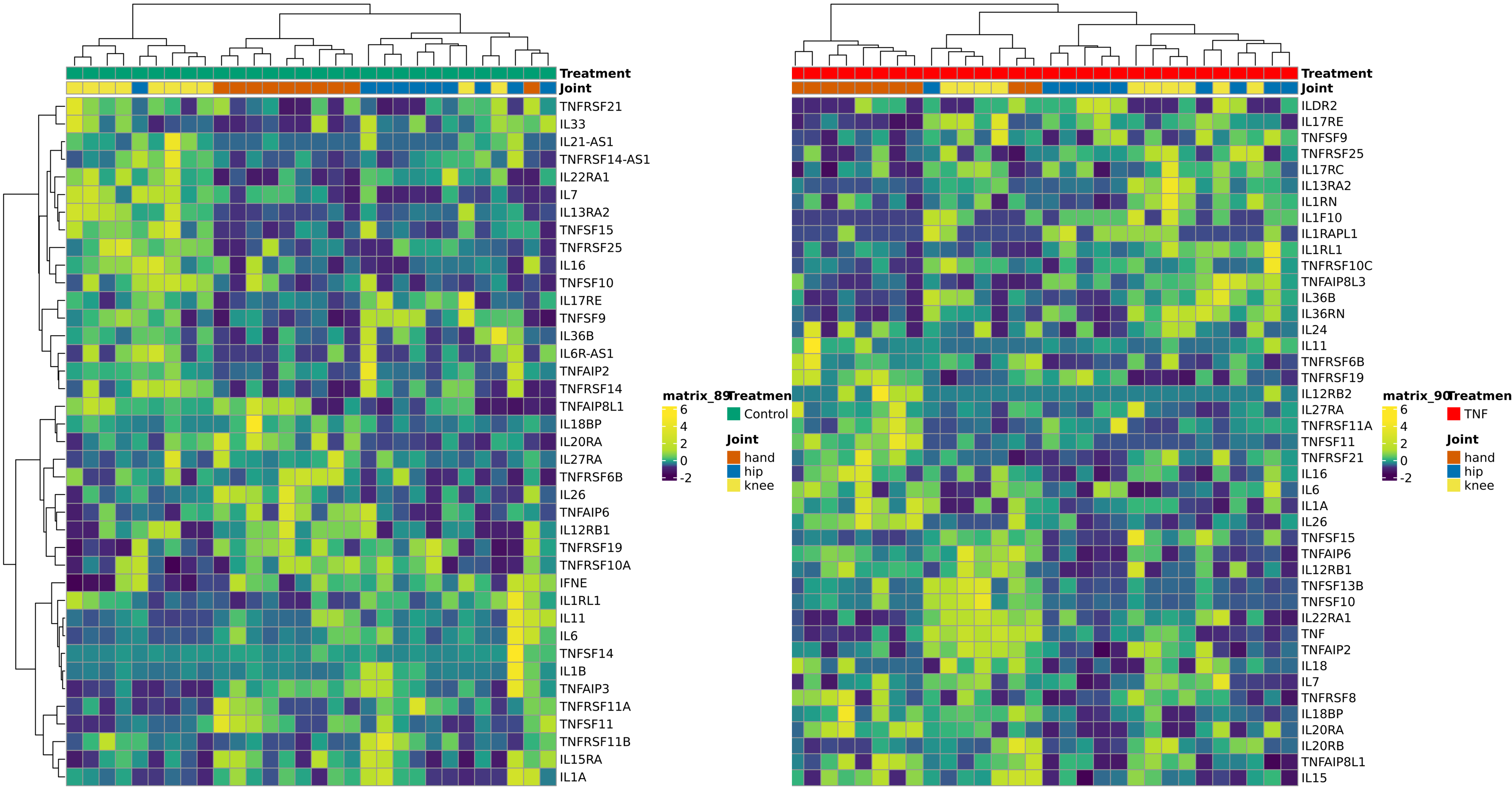

S2. Unsupervised clustering using differentially expressed cytokines. Heatmap using unsupervised clustering of differentially expressed cytokines within medium (left) or TNF-stimulated (right) conditions. Ward's Hierarchical Agglomerative Clustering Method using the correlation distance was used to cluster samples by cytokine expression. Unstimulated (left) and TNF-stimulated (right) FLS exhibit the greatest mixing with hip and knee.

S3. Unsupervised clustering using differentially expressed limb patterning genes

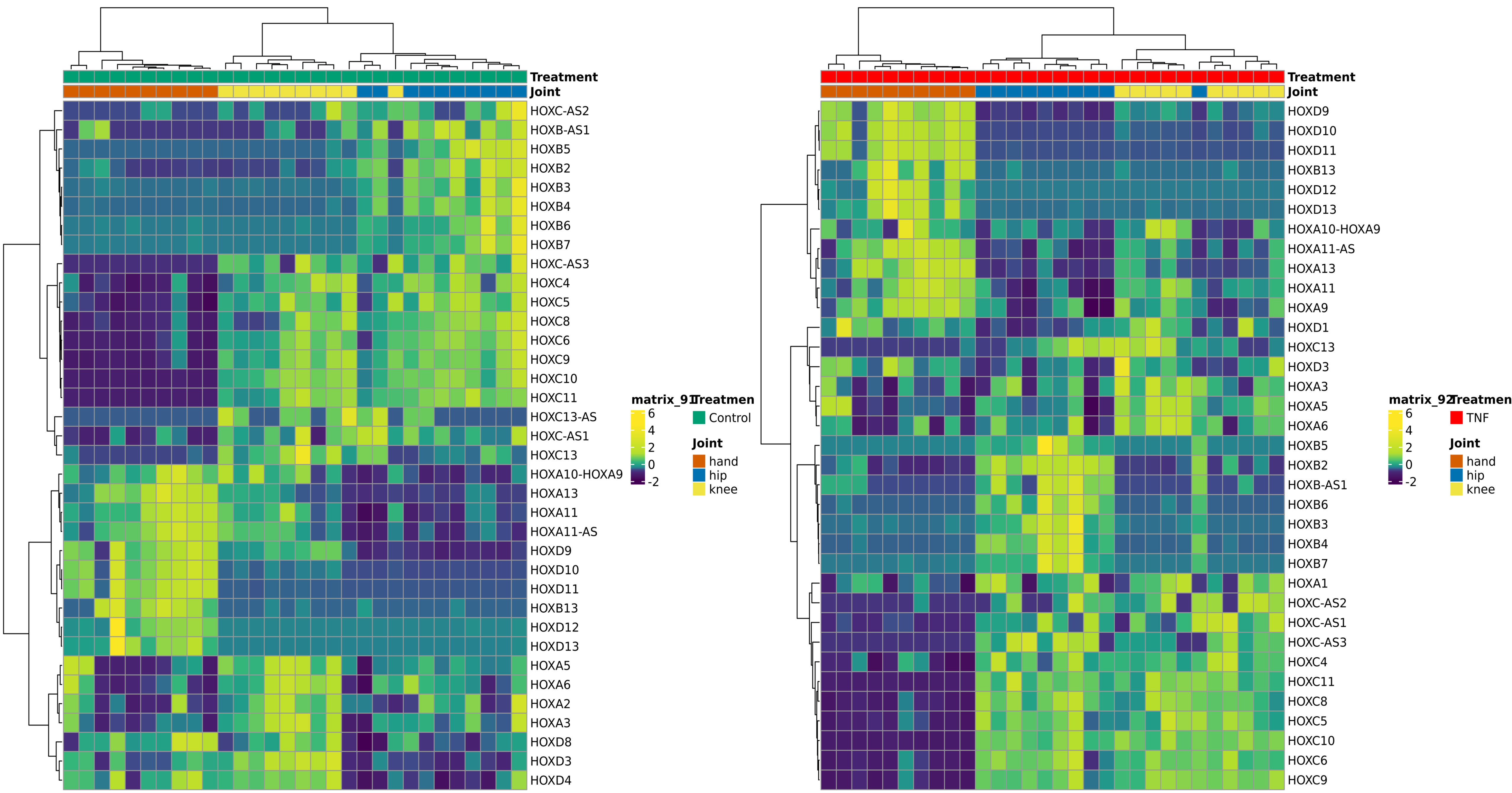

S3. Unsupervised clustering using differentially expressed limb patterning genes. Hierarchical clustering within unstimulated or TNF-stimulated FLS (ward.D2, correlation method). Heatmap using unsupervised clustering of differentially expressed HOX genes within unstimulated (left) or TNF-stimulated (right) conditions. Consistent expression patterns between hip with knee were observed whereas hand FLS had the most distinct expression compared with the other joints.

S4. HOTAIR chromatin accessibility and gene expression plots

A **B**

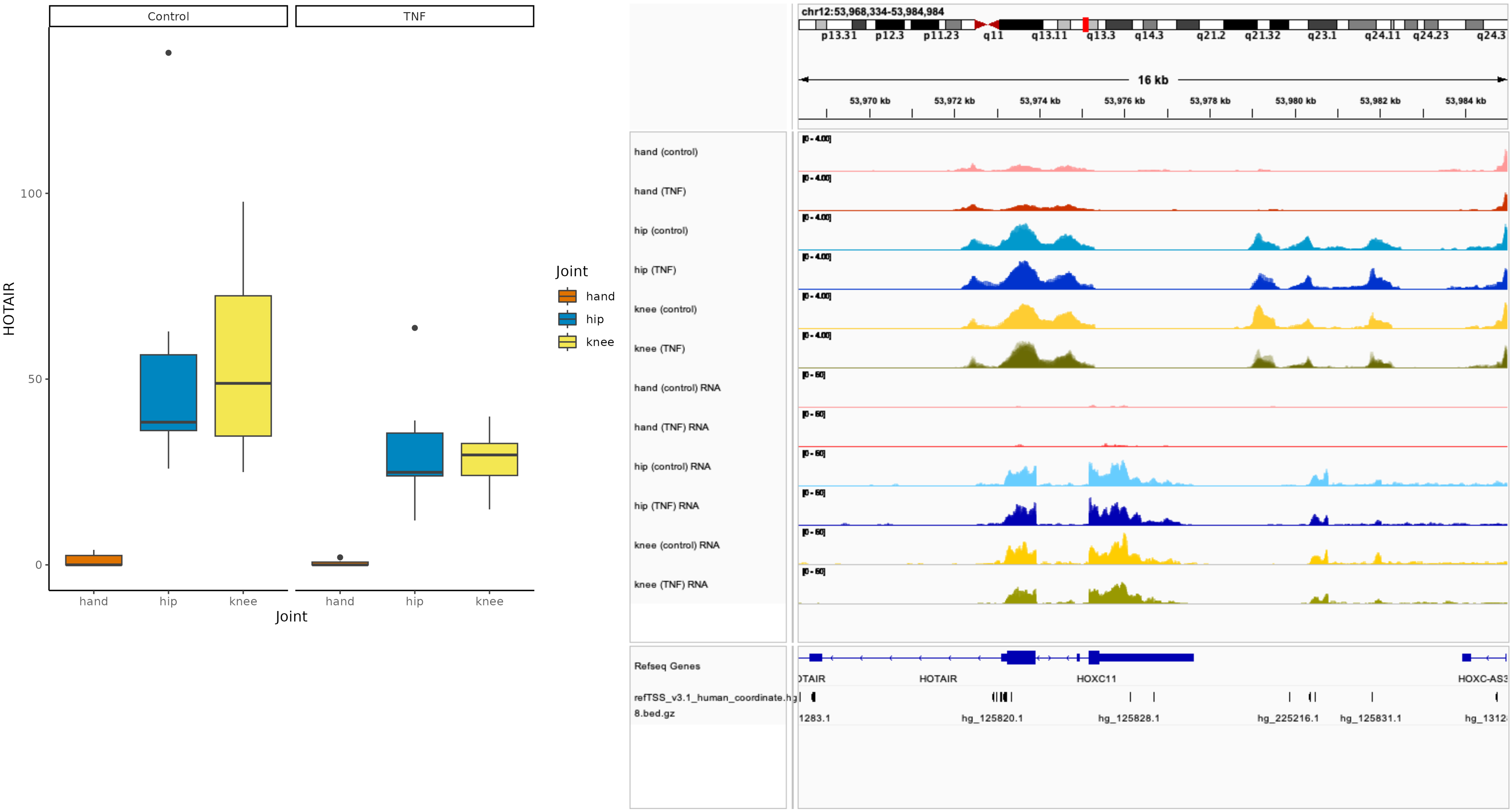

S4A. Box plot of gene expression profiles of HOTAIR within treatment and between joints. View **S5** for significance values

B. . HOTAIR chromatin accessibility and gene expression plots. Genome browser of chromatin accessibility (top 6, ordered by joint and treatment) and gene expression (bottom 6, ordered by joint and treatment). View **S6** for significance values.

S5. HOTAIR gene expression tables

HOTAIR gene expression profiles

comparison	gene	pval	FC	Treatment	feature
knee_hand	HOTAIR	1.49e-04	26.038	Control	HOTAIR
hip_hand	HOTAIR	1.49e-04	24.909	Control	HOTAIR
knee_hip	HOTAIR	6.31e-01	1.045	Control	HOTAIR
knee_hand	HOTAIR	1.32e-04	21.015	TNF	HOTAIR
hip_hand	HOTAIR	1.32e-04	21.842	TNF	HOTAIR
knee_hip	HOTAIR	9.71e-01	0.962	TNF	HOTAIR

S5. HOTAIR gene expression tables. HOTAIR gene expression significance values. FC is first joint / second joint. For example, TNF knee_hand has FC of 21.01. This means knee has higher HOTAIR expression than hand by 21.01.

S6. HOTAIR chromatin accessibility tables

chromatin accessibility of HOTAIR promoters (<1kb TSS)

comparison	chrom	start	end	FDR	FC	Treatment
hand_hip	chr12	53973686	53974086	2.0e-80	-2.430	Control
knee_hand	chr12	53973686	53974086	2.4e-74	2.357	Control
knee_hip	chr12	53973686	53974086	7.4e-01	-0.060	Control
hand_hip	chr12	53973593	53973993	4.35e-88	-2.438	TNF
knee_hand	chr12	53973593	53973993	2.69e-84	2.399	TNF
knee_hip	chr12	53973593	53973993	9.06e-01	-0.025	TNF

S6. HOTAIR chromatin accessibility tables. HOTAIR promoter chromatin accessibility significance values within treatment and between joints. FC is first joint / second joint. For example, TNF knee_hand has FC of 2.4. This means knee has a higher peak than hand by 2.4.

S7. PCA of chromatin accessibility features including wrist meta labels within unstimulated (left) and TNF (right) stimulated FLS.

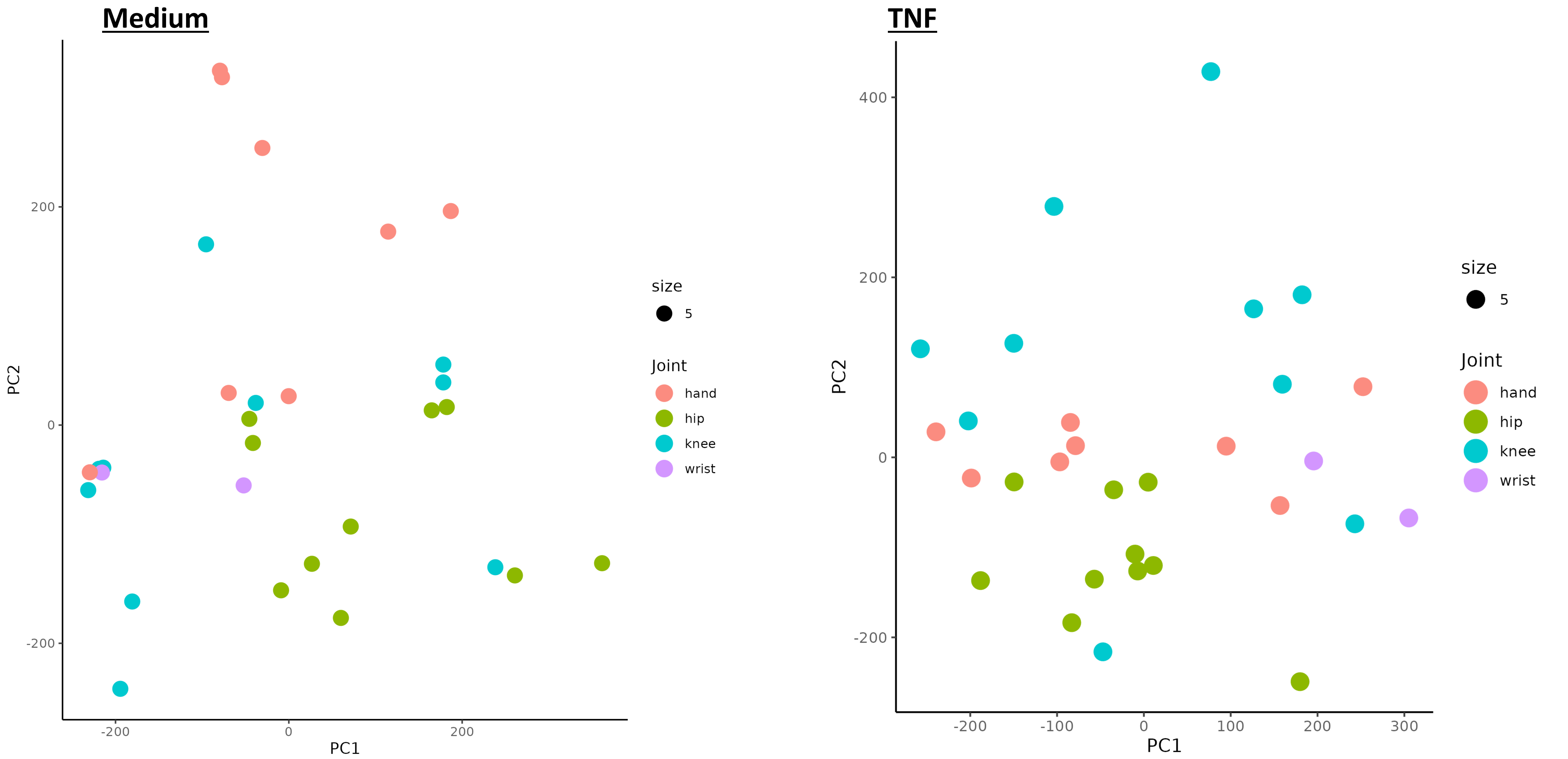

S7. PCA of chromatin accessibility features including wrist meta labels within unstimulated (left) and TNF-stimulated (right) FLS. To evaluate joint-specific differences in chromatin accessibility, PCA using sub-categorical meta labels were used. We did not observe any stratification between wrist vs CMC/MCP/PIP samples using epigenetic features.

S8. Unstimulated FLS have little overlap between DEGs and DARs

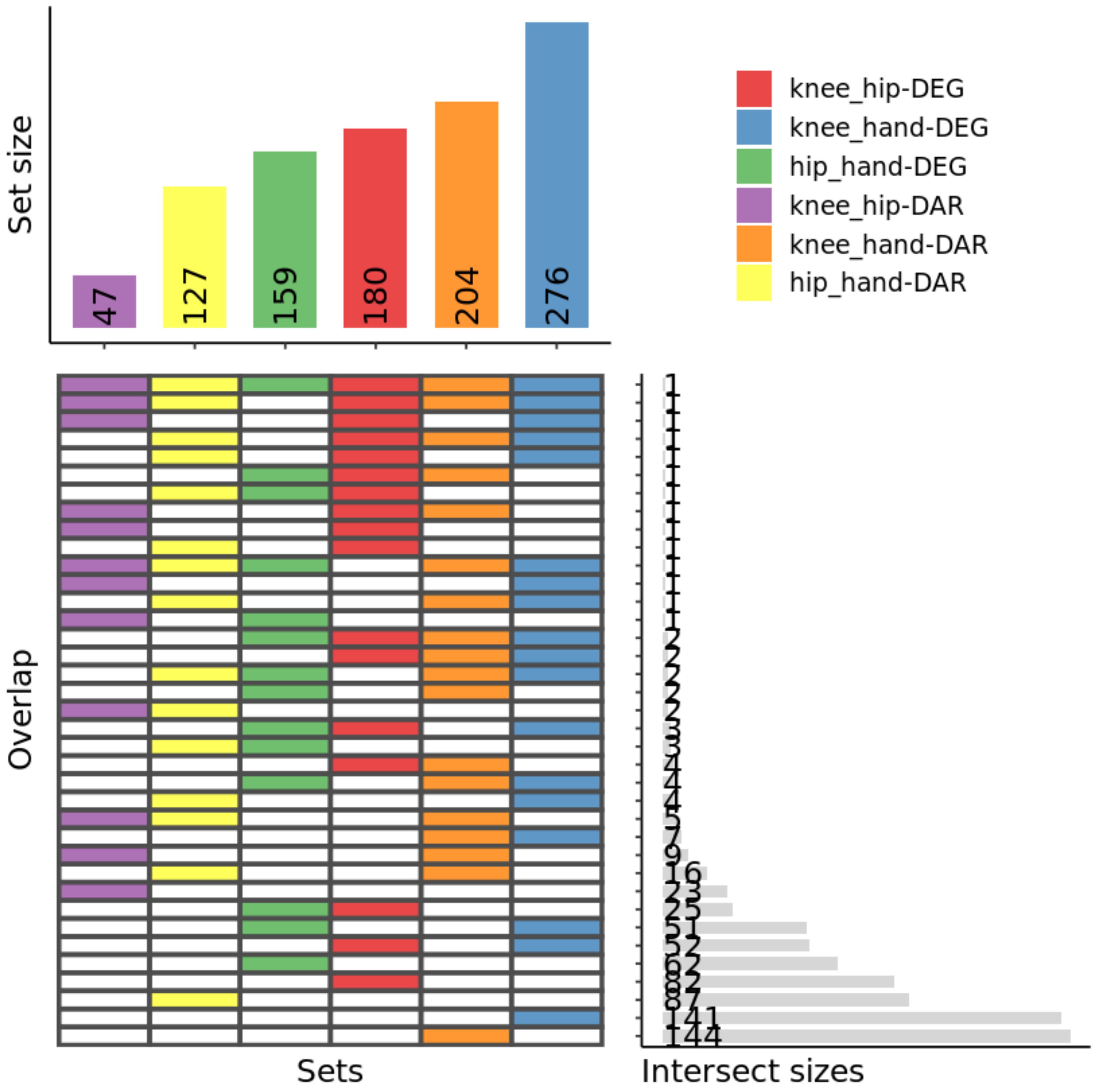

S8. Unstimulated FLS have little overlap between DEGs and DARs . Upset plot that visualizes the overlap of DARs and DEGs for each pairwise comparison within unstimulated FLS and between hand, hip, and knee. This is read identical to Figure 2B and Figure 3B. The top bars indicate number of all features for that group. The horizontal bars indicate the number of features within that overlap subsection. For example, hand vs hip DARs (yellow) and DEG (green) have very few overlapping features suggesting FLS are in an epigenetically poised state.

S9. HOTAIR transcriptional network identifed from Taiji

A. HOTAIR network (Control)

B. HOTAIR network (TNF)

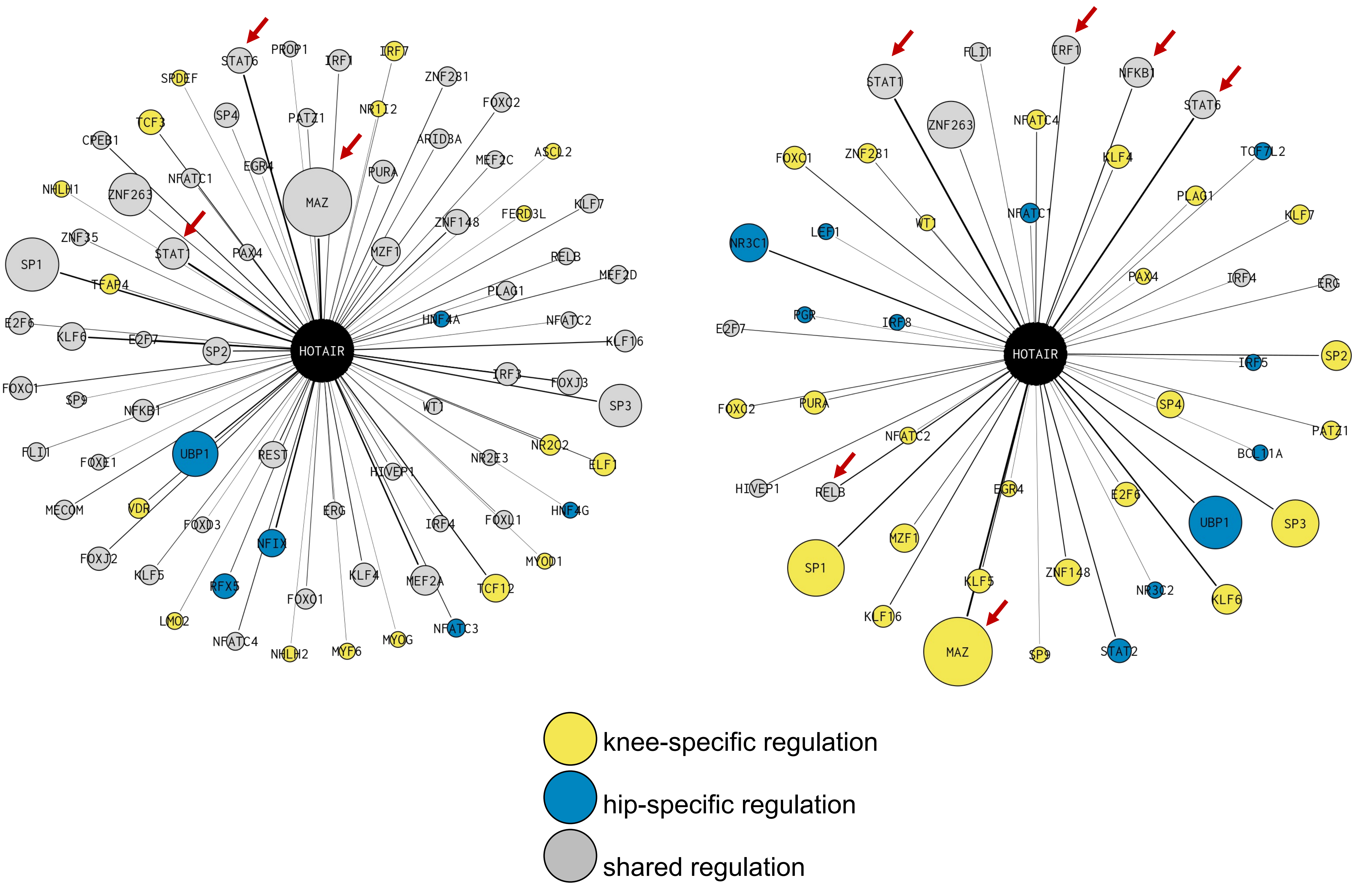

S9. HOTAIR transcriptional network identifed from Taiji . For each treatment (Control, TNF), we identified joint-specific and shared regulation of HOTAIR. Nodes (circles) are scaled by PageRank (higher PageRank → bigger node). Nodes are colored blue (hip-specific), yellow (knee-specific), or grey if the regulatory relationship is shared by both hip and knee. If the node is joint-specific (i.e. blue or yellow color), the PageRank size is scaled by average PageRank of that joint. If the node is shared (i.e. grey), the PageRank size is scaled by the average PageRank across knee and hip. Edges are weighed by the average edge weight (same method as node weight). The darker the edge, the higher the edge weight and thus the greater the regulatory potential between that TF to HOTAIR. Key TFs are indicated with red arrows.

S10. Hand FLS has significant enrichment of genes associated with the ‘activated’ state (DEGs)

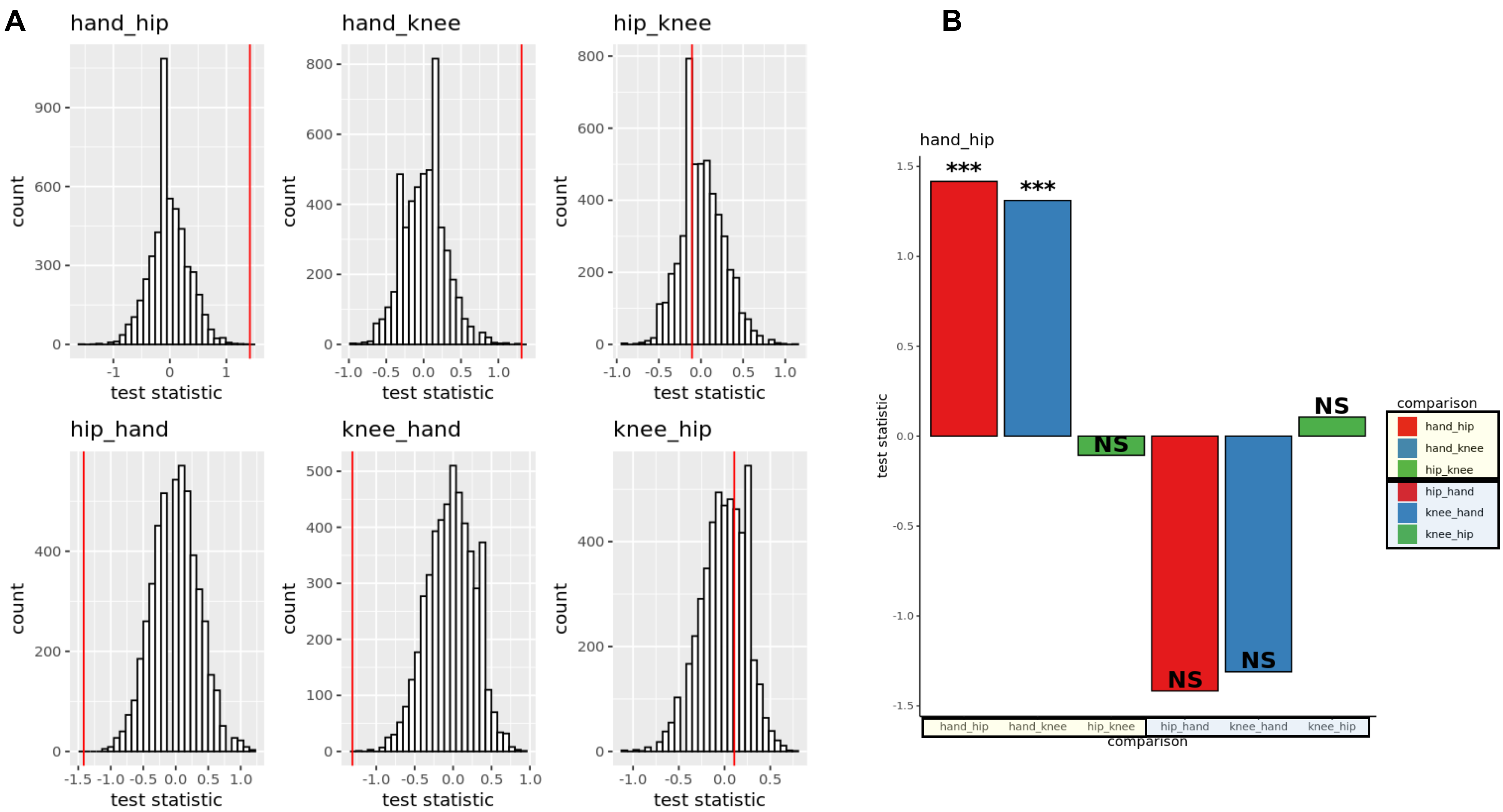

S10. Hand FLS has significant enrichment of genes associated with the ‘activated’ state (DEGs). Significant enrichment of activation markers between joints *i* and *j* are identified using a permutation test by randomly shuffling the joint labels with the greatest expression (defined by the median for each joint) of that activated/resting marker and then recalculating the ratio of max #activated to #resting markers.

Figure **S10A** is the histogram of test statistics of shuffled activated/resting marker labels. The vertical line is the original test statistic. For example, in hand_hip plot, we hypothesized that hand would have a greater ratio of activated to resting marker expression. The original test statistic > 1 indicating hand FLS has greater enrichment of activated markers compared to hip. P-value < 0.05 indicates this is unlikely due to chance, as shown in **S10B**. Though knee has the greatest expression of the most activated markers, it is attenuated by increased expression of resting markers**. *, **, ***** represents p-value < 0.05, 0.01, and 0.005, respectively.

S11. Hand FLS has significant enrichment of genes associated with the ‘activated’ state (all markers)

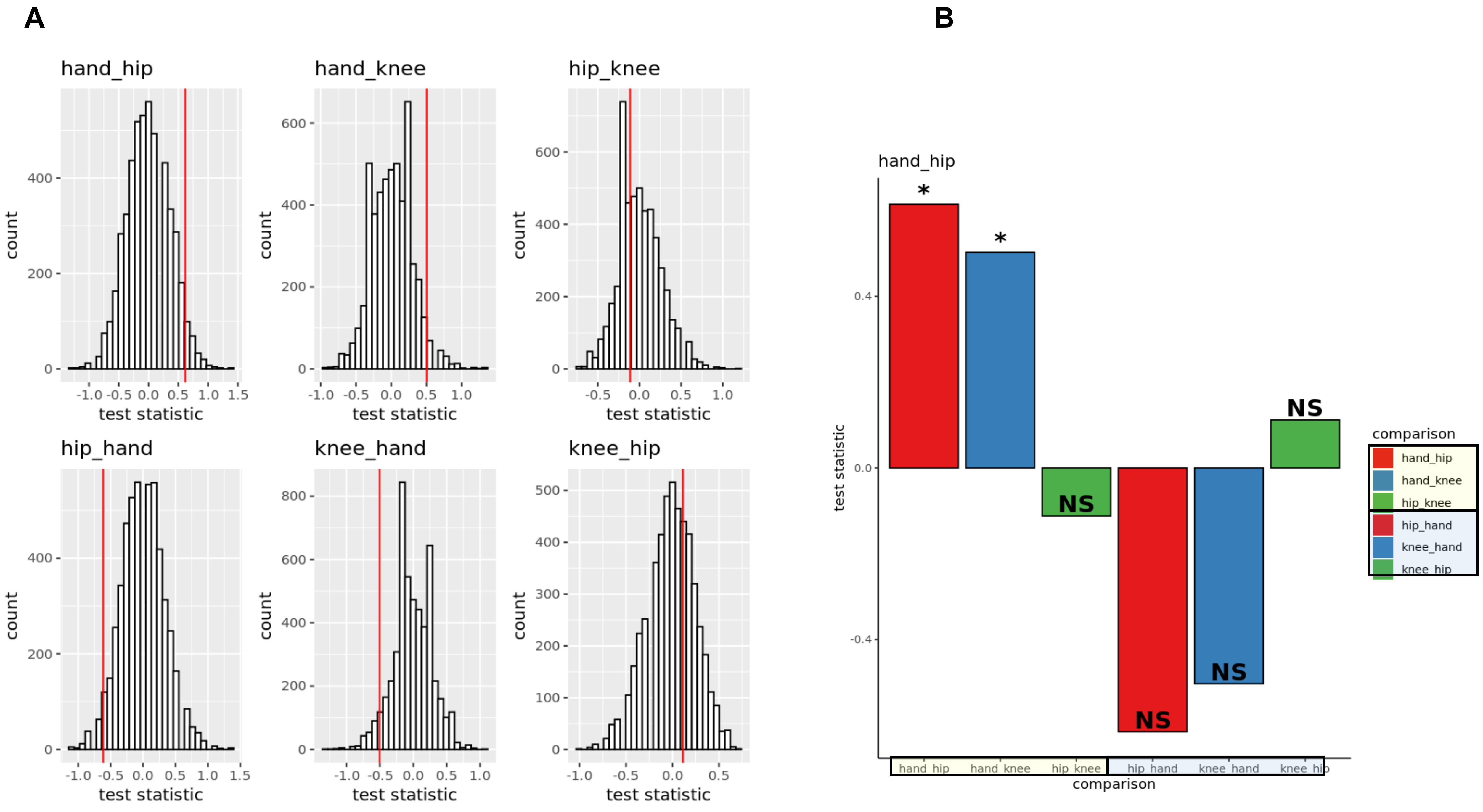

S11. Hand FLS has significant enrichment of genes associated with the ‘activated’ state (all markers). Activated and resting marker expression were evaluated using all markers within unstimulated FLS. Similar observation using differentially expressed markers are observed when using all markers with the greatest activated marker expression observed in hand FLS. ***, **, ***** represents p-value < 0.05, 0.01, and 0.005, respectively. See **S10** for more detailed description.

S12. TNF-stimulated hand FLS has increased burden of cytokine and MMP expression (DEGs)

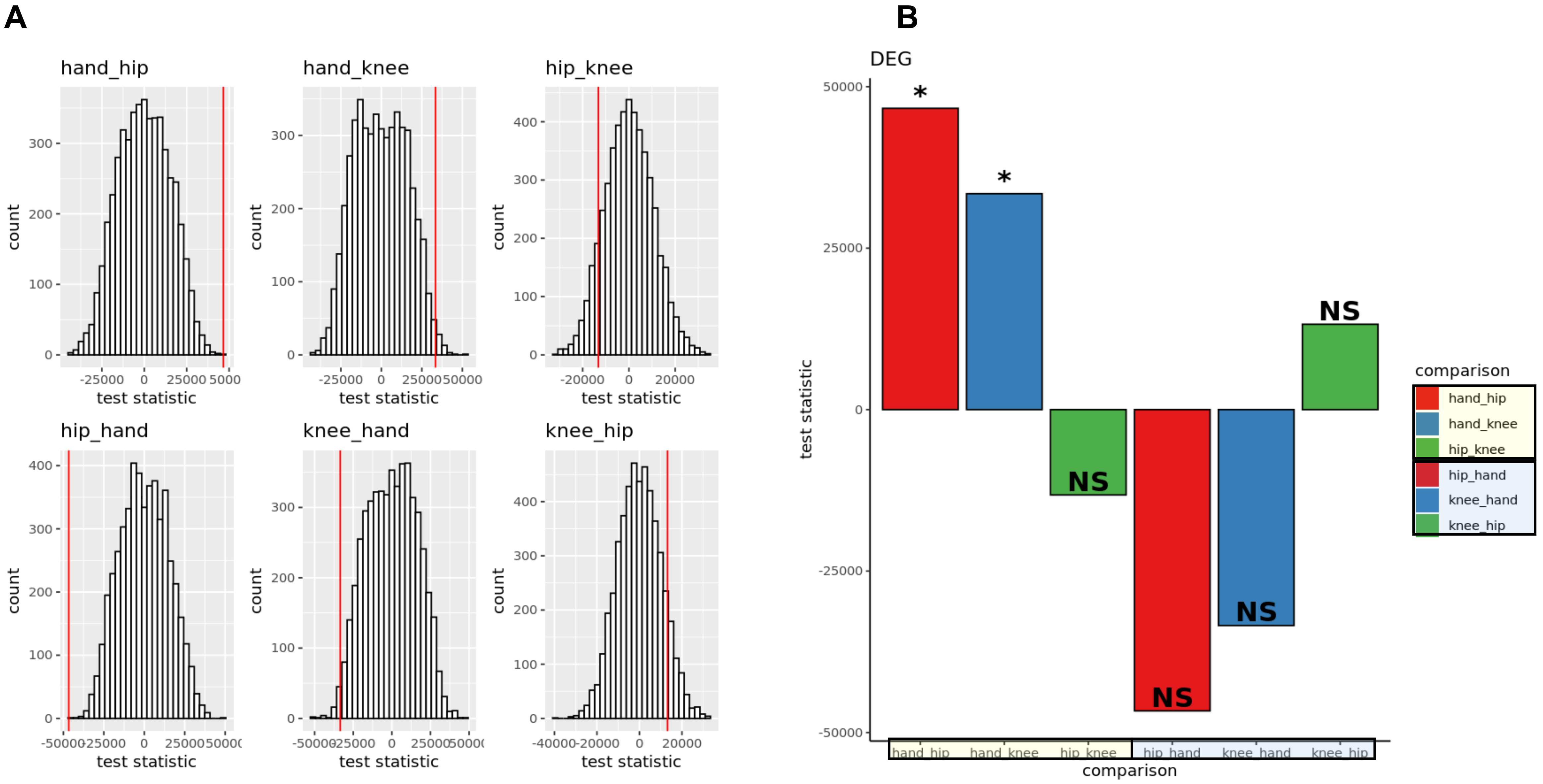

S12. TNF-stimulated hand FLS has increased burden of cytokine and MMP expression (DEGs). We identified the enrichment of cumulative cytokine and MMP expression in TNF-stimulated cell-lines by taking the cumulative expression of cytokines and MMPs averaged (by median) per joint. For each pairwise comparison, significant upregulation of cytokines between joints *i* and *j* are identified using a permutation test by randomly shuffling the joint labels and taking the sum expression.

Figure **S12A** is the histogram of test statistics of shuffled joint labels within TNF-stimulated conditions given differentially expressed cytokines. The vertical line is the original test statistic. For example, in hand_hip plot, we hypothesized that hand would have a greatest cumulative expression of cytokines compared to hip. The original test statistic > 1 indicating hand FLS has greatest cumulative expression of cytokines. P-value < 0.05 indicates this is likely not due to chance. ***, **, ***** represents p-value < 0.05, 0.01, and 0.005, respectively.

S13. TNF-stimulated hand FLS has increased burden of cytokine and MMP expression (all markers)

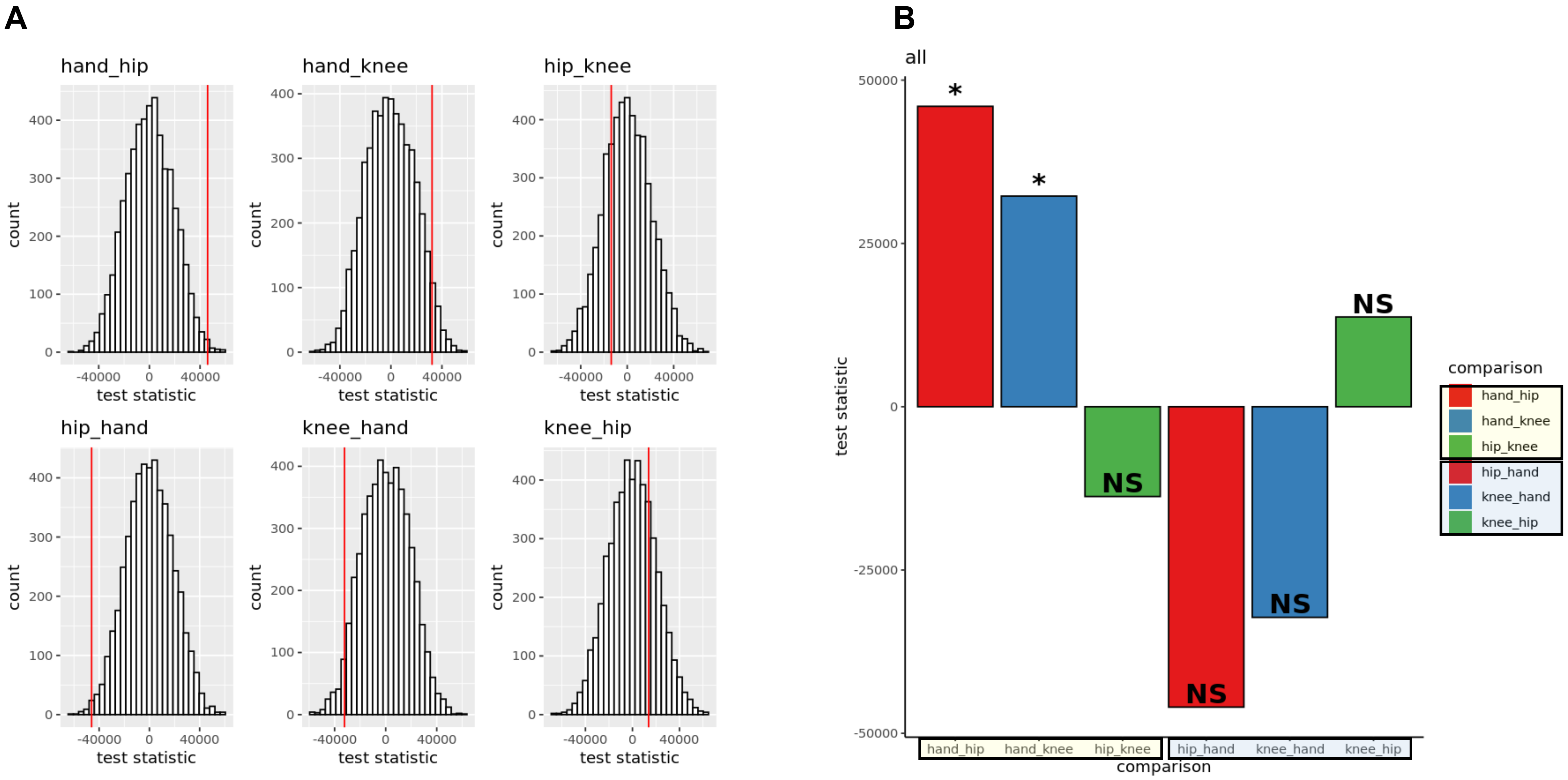

S13. TNF-stimulated hand FLS has increased burden of cytokine and MMP expression (all markers). Cumulative cytokine and MMP expression were evaluated using all cytokines within unstimulated FLS. Similar observation using differentially expressed cytokines are observed when using all markers with the greatest cumulative expression observed in hand. ***, **, ***** represents p-value < 0.05, 0.01, and 0.005, respectively. View **S12** for a more detailed description.

S14. Batch effects are not observed in ATACseq experiments

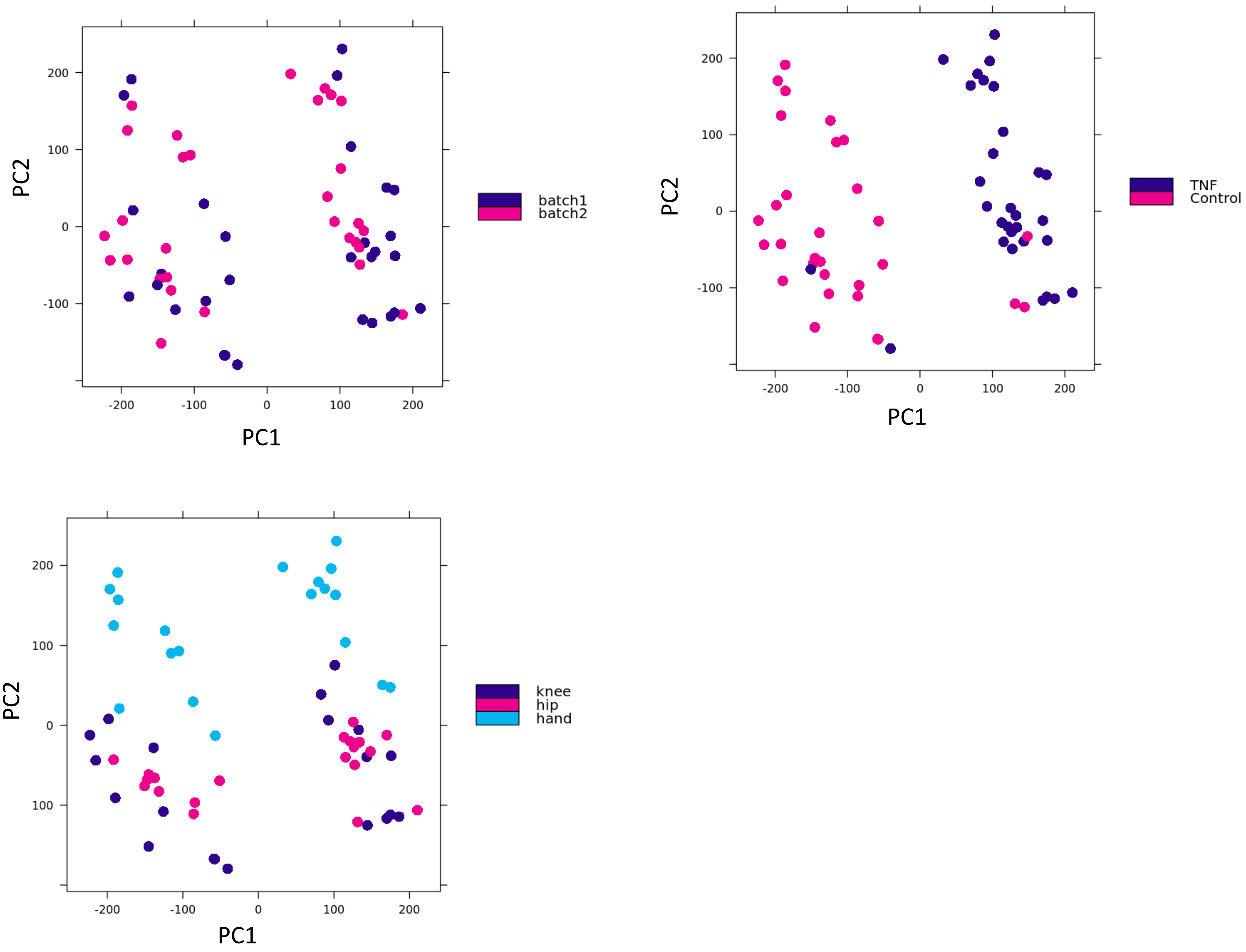

S14. Batch effects are not observed in ATACseq experiments. ATAC-seq files were processed in two batches. Batch effects were evaluated using PCA. Samples segregate by condition (control, TNF) and joint (hand, hip, knee). Batch effects were not observed. NOTE: RNA-seq files were processed in the same batch.

S15. Taiji workflow

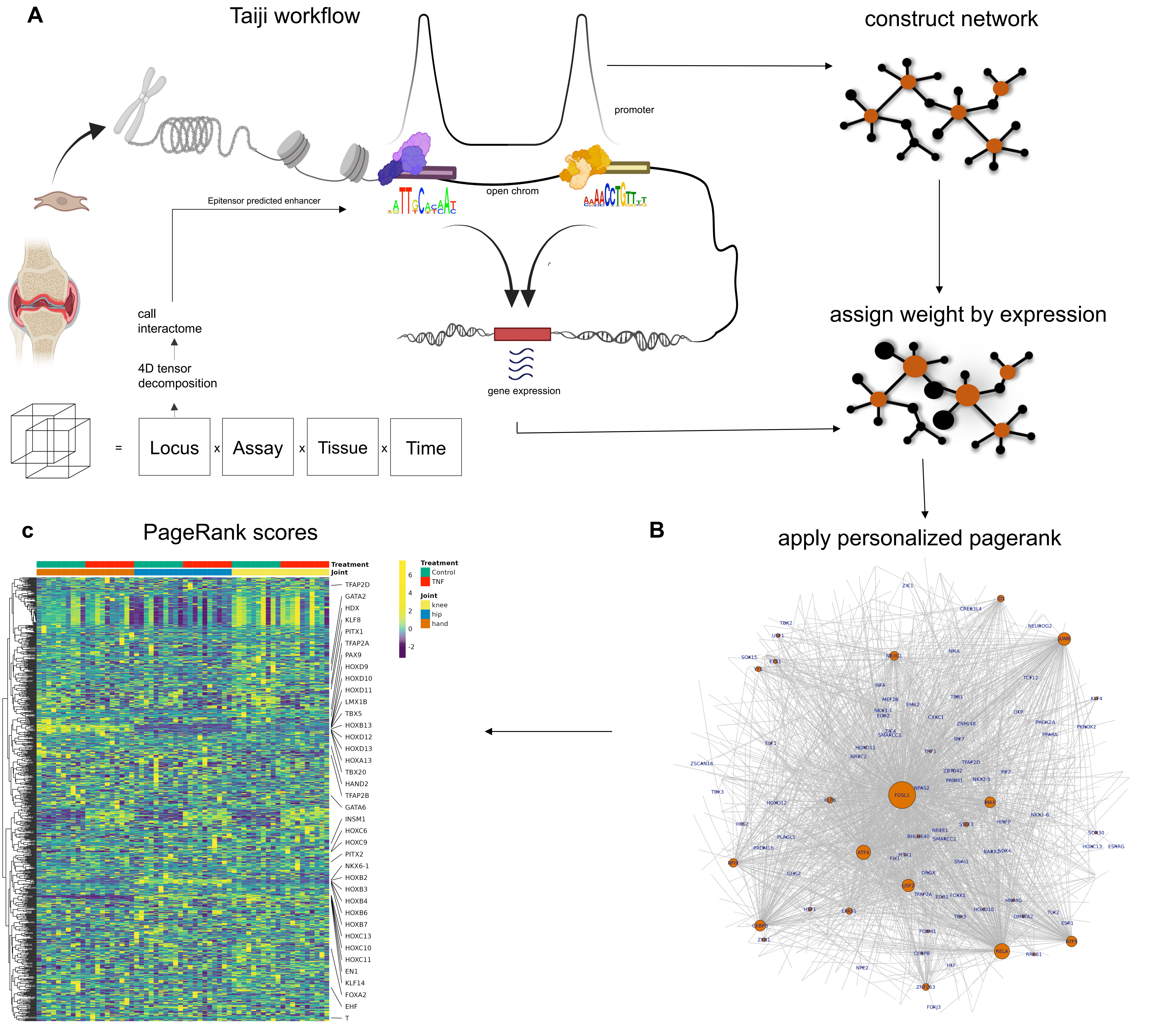

S15. Taiji workflow . Schematic of the taiji workflow. Taiji integrates RNA-seq, ATAC-seq, and enhancer-promoter interactions (the top 10% most confident predictions from Epitensor[8]). For each sample, Taiji produced a genetic network and inferred TF importance via personalized PageRank calculations for 745 TFs with known motifs. Briefly, the nodes in the network are genes and are weighted by the normalized gene-expression level. To create edges between regulators and regulatees, open chromatin promoter and enhancer regions are queried for TF motifs documented in the Cis-BP[9] database. These edges are weighted by the motif score reflecting the binding affinity, TF expression levels, and target open chromatin peak intensity. The personalized PageRank algorithm is then applied to the resulting directional and weighted network to determine relative importance of each TF for each sample. The output of the Taiji workflow for each sample are the personalized PageRank scores for 745 TFs, the network topology consisting of directional regulator—regulatee relations with each pair's associated edge weight and node weights.
